# Recent Advances on Nanoparticle based Strategies for Improving Carotenoid Stability and Biological Activity

**DOI:** 10.3390/antiox10050713

**Published:** 2021-04-30

**Authors:** Kandi Sridhar, Baskaran Stephen Inbaraj, Bing-Huei Chen

**Affiliations:** Department of Food Science, Fu Jen Catholic University, New Taipei City 24205, Taiwan; sridhar4647@gmail.com (K.S.); or 138547@mail.fju.edu.tw (B.S.I.)

**Keywords:** nanoencapsulation, carotenoid, in vitro release, antioxidant activity, bioaccessibility, bioavailability, biological activity

## Abstract

Carotenoids are natural pigments widely used in food industries due to their health-promoting properties. However, the presence of long-chain conjugated double bonds are responsible for chemical instability, poor water solubility, low bioavailability and high susceptibility to oxidation. The application of a nanoencapsulation technique has thus become a vital means to enhance stability of carotenoids under physiological conditions due to their small particle size, high aqueous solubility and improved bioavailability. This review intends to overview the advances in preparation, characterization, biocompatibility and application of nanocarotenoids reported in research/review papers published in peer-reviewed journals over the last five years. More specifically, nanocarotenoids were prepared from both carotenoid extracts and standards by employing various preparation techniques to yield different nanostructures including nanoemulsions, nanoliposomes, polymeric/biopolymeric nanoparticles, solid lipid nanoparticles, nanostructured lipid nanoparticles, supercritical fluid-based nanoparticles and metal/metal oxide nanoparticles. Stability studies involved evaluation of physical stability and/or chemical stability under different storage conditions and heating temperatures for varied lengths of time, while the release behavior and bioaccessibility were determined by various in vitro digestion and absorption models as well as bioavailability through elucidating pharmacokinetics in an animal model. Moreover, application of nanocarotenoids for various biological applications including antioxidant, anticancer, antibacterial, antiaging, cosmetics, diabetic wound healing and hepatic steatosis were summarized.

## 1. Introduction

Carotenoids are colored natural pigments belonging to a large family of C_40_ skeleton with eight isoprene molecules [1]. They are classified into xanthophylls and carotenes with the former such as lutein, β-cryptoxanthin and astaxanthin containing one or more oxygen atoms, while the latter such as α- carotene and β-carotene, lycopene and phytoene consisting of hydrogen and carbon atoms [2]. Carotenoid-rich foods have received great attention in human health due to their physiological functions such as antioxidant and anti-cancer as well as the ability to prevent chronic diseases such as age-associated macular degeneration and cardiovascular disease [3,4]. It has been well demonstrated that the functional properties of carotenoids were associated with their chemical structure i.e., the number of conjugated double bonds and the presence of different kinds of end-groups. However, these structural properties are also responsible for the carotenoid’s instability to light, high temperature, oxygen and metal ions, resulting in high susceptibility to oxidation and low bioavailability [3].

Given the multiple health benefits of carotenoids, they are widely used as a natural colorant and antioxidant in both pharmaceutical and food industries for prolonging shelf-life in dairy, meat, confectionary and beverage products [2]. However, carotenoids may undergo loss in functional properties during food processing owing to their instability and interaction with other food ingredients. Also the presence of digestive enzymes and some other nutrients in vivo as well as pH can alter carotenoid stability [4]. Consequently, it is vital to develop novel techniques to prevent carotenoid degradation for enhancement of bioavailability and bioactivity. Over the past decade, micro- and/or nano-encapsulation have emerged as imperative techniques for formulating food-based carotenoid carriers with improved physicochemical property and release behavior, as well as for prolonging blood circulation and efficient cellular uptake [2,5]. Comparatively, the microencapsulation technique fails to produce nanoparticles that are capable of penetrating into deeper portions of specific organs and tissues, resulting in poor bioavailability and bioactivity in vivo [6,7]. Thus, the transformation from microencapsulation to nanoencapsulation plays a pivotal role in reducing particles to nanosize by employing either top-down or bottom-up methods [8]. 

**Figure 1:**
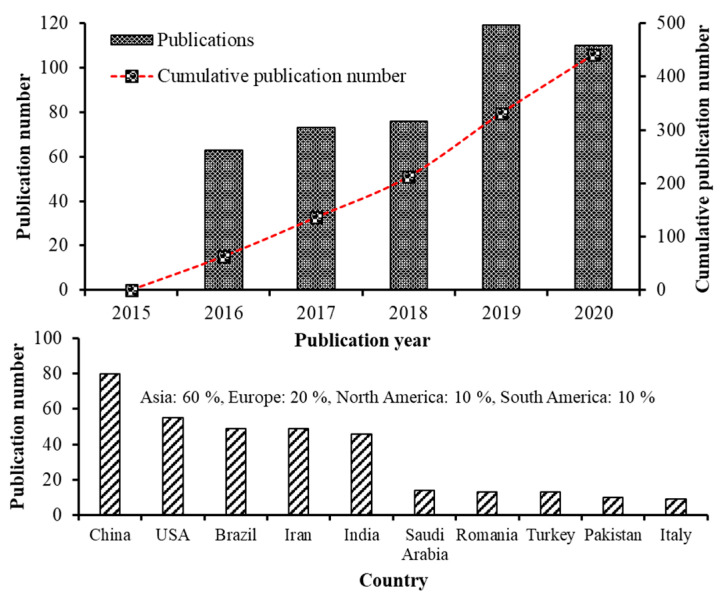
Research on carotenoid nanoemulsions over the last 5 years. The number of publications and global distribution. Source: Web of Science^™^.

Recent advancements in the field of nanoscience and nanotechnology have enabled preparation of nanoscale functional compounds by encapsulating into a wide variety of nanostructures including nanoemulsions (NEs), nanoliposomes (NLs), nanocapsules (NCs), nanofibers (NFs), nanoparticles (NPs), solid lipid nanoparticles (SLNPs), nanostructured lipid carriers (NLCs) and supercritical fluid-based nanoparticles [4,5,9]. Based on the bibliometric search conducted in the Web of Science database (version 5.34) using keywords such as carotenoid nanoemulsion, carotenoid nanoparticle, carotenoid nanoencapsulation, carotenoid nanoliposome, carotenoid liposome, carotenoid micelle and carotenoid dispersion, the number of articles published from 2015-2020 was 441, of which the publications from 2019–2020 was higher (229) in terms of publication rate compared to that published between 2015–2018 (212). This trend is in line with the previous bibliometric studies [10,11]. Moreover, of the top 10 countries listed on research publications in carotenoid nanoencapsulation (2015–2020), China showed the highest publication output (23.7%) followed by USA (16.3%), Brazil (14.5%), Iran (14.5%), India (13.6%), while the other five countries including Saudi Arabia, Romania, Turkey, Pakistan and Italy, accounting for 17.4%, with an overall contribution from Asia, Europe and Americas being 60, 20 and 20%, respectively (Figure 1). Further analysis on difference in research characteristics among the top five highly-contributed countries during 2015–2020 showed that China (30) dominated with studies related to preparation and stability evaluation of nanocarotenoids, followed by Iran (20), India (19), USA (17) and Brazil (13). Likewise, most nanocarotenoid studies dealing with in vitro gastrointestinal release and bioavailability were from China (20), followed by USA (19), Iran (18), India (12) and Brazil (10). Many studies have also focused on fortification of nanoencpasulated carotenoids in a wide range of functional foods in dairy, bakery, and confectionary industries over last five years, with the top five countries accounting for 2.8–9.3% of total nanocarotenoid publications. For in vivo studies, although there was less publications by top 5 countries (5–22), a significant research output is apparent. Notably, China remained on the top with 22 publications dealing with determination of bioavailability and bioaccessibility, followed by USA (14), Iran/India (10 each) and Brazil (5). This highlights the need for many in vivo studies for proof-of-concept, functional validation, utility and clinical relevance of nanocarotenoids, which can be attained through promoting collaborative researches among institutes and countries for translation of nanocarotenoids into a botanic drug.

Reported studies on nanocarotenoids were mainly dealing with formulation of nanosized carotenoid carriers by nanotechniques, characterization and stability evaluation as well as determination of in vitro release behavior, bioaccessibility, bioavailability and biological activity [5]. Herein, we intend to overview the recent advancements in carotenoid nanoencapsulation techniques reported within the last five years, with emphasis on their preparation methods, physicochemical characteristics, in vitro release behavior, stability, bioavailability and biological activity.

## 2. Carotenoid Biosynthesis and Stability-Overview

**Figure 2:**
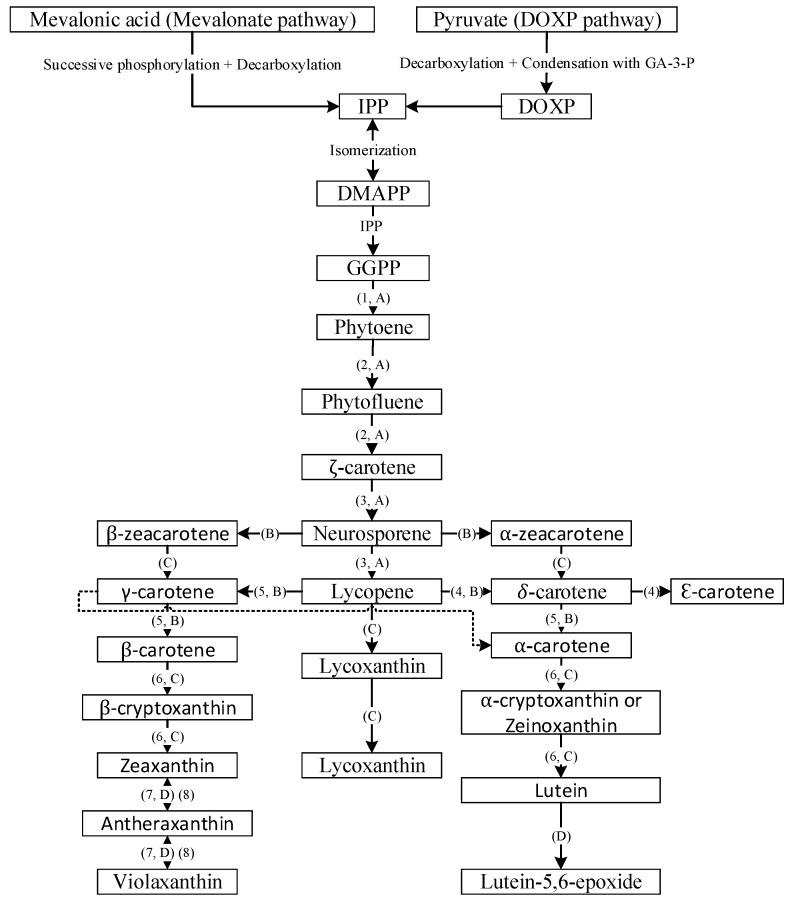
General overview of carotenoid biosynthesis pathway. DOXP = 1-deoxy-D-xylulose, GA-3-P = glyceraldehyde 3-phosphate, IPP = isopentyl diphosphate, DMAPP = dimethylallyl diphosphate and GGPP = geranylgeranyl pyrophosphate; 1 = phytoene synthase, 2 = phytoene desaturase, 3 = ζ-carotene desaturase, 4 = lycopene e-cyclase, 5 = lycopene β-cyclase; 6 = β-carotene hydroxylase, 7 = zeaxanthin epoxidase and 8 = violaxanthin de-epoxidase; A = desaturation, B = cyclization, C = hydroxylation and D = epoxidation.

Carotenoids, a class of isoprenoids, are formed by the C_5_ building units of isopentenyl diphosphate and dimethylallyl diphosphate, obtained separately by two different pathways including the mevalonate (MVA) and the 2-C-methyl-D-erythritol 4-phosphate (MEP) pathways [12]. The isopentenyl diphosphate undergoes isomerization to yield dimethylallyl diphosphate, which further condenses with another molecule of isopentenyl diphosphate to yield C_20_ geranylgeranyl pyrophosphate. Then the two molecules of geranylgeranyl pyrophosphate combine with each other to yield the first carotenoid molecule phytoene (C_40_) and sequential incorporation of double bonds at alternate positions of phytoene, resulting in formation of phytofluene, ζ-carotene, neurosporene and lycopene (Figure 2) [13]. Through branched cyclization of lycopene, carotenoids with one β-ring and one ε-ring (e.g., α-carotene and lutein) and two β-rings (β-carotene, zeaxanthin and antheraxanthin) are produced. Further advancement of carotenoid synthesis occurred through attachment of oxygen moieties to hydrocarbon carotenoids such as α-carotene and β-carotene for formation of xanthophylls (Figure 2) [13].

The presence of long-chain conjugated double bonds in carotenoids makes them highly susceptible to degradation under acid, light and high temperature conditions [3]. For instance, carotenoids were shown to degrade at a faster rate in the presence of light through generation of singlet oxygen that eventually binds with the hydrocarbon chain in carotenoids leading to degradation [14]. More recently, two theories have been proposed for oxidative degradation of carotenoids, namely random and central cleavage theories, with oxidation occurring randomly at different sites or at the central bond of a carotenoid molecule, respectively [15]. In a study dealing with thermal degradation of lutein and β-carotene, Giménez, et al. [16] reported a progressive increase in degradation following a rise in heating temperature from 30–90 °C. Also, β-carotene could undergo degradation to form epoxides and carbonyl compounds (apocarotenals) via a free radical reduction mechanism [13]. Thus, owing to the instability of carotenoids caused by multiple factors, it is important to develop appropriate strategies for preventing degradation, prolonging shelf-life and enhancing bioavailability of carotenoids.

## 3. Conventional Microencapsulation vs. Nanoencapsulation

Microencapsulation of unstable and water-insoluble bioactive compounds such as carotenoids involves trapping them within a special coating material for preparation of micron-sized particles with a mean size ranging from 1 to 500 µm. Micronized spherical particles are capable of controlling both loading and releasing of bioactive compounds [17]. The conventional microencapsulation process can be broadly classified into three categories depending on how microparticles are prepared including chemical, physicochemical and physicomechanical processes [18,19]. For example, Polyakov and Kispert [20] reviewed a carotenoid inclusion complex (e.g., β-carotene enriched inclusion complex) with polysaccharides including arabinogalactan, cyclodextrin and glycyrrhizin, and demonstrated increased stability and bioavailability compared to free carotenoids, while García, et al. [21] reported an enhanced thermal stability (up to 100 °C) of spherical microcapsules produced from carotenoid-rich mango, banana and tamarillo powders by spray-drying with maltodextrin. Likewise, several studies have demonstrated the ability of microencapsulated carotenoids to improve physicochemical characteristics, storage stability and bioavailability for further development into value-added functional foods [22,23,24,25,26]. Several microencapsulation techniques used for enhancement of carotenoid stability and bioavailability have been reviewed by Soukoulis and Bohn [2]. 

Although the microencapsulation techniques are efficient, the recent clean labelling trends have prevented the use of dairy, lactose, sugar, sodium, gluten, fats and carbohydrate as coating material, thus further limiting the choice of suitable encapsulation materials [27]. In addition, the most commonly used encapsulant maltodextrin possesses low emulsifying ability thereby reducing the encapsulation efficiency (EE) [28]. More recently, Sun, et al. [6] pointed out that the average size of microcapsules is a critical parameter which can significantly affect the physicochemical characteristics, stability, sensory property, bioavailability and release behavior. Also, micron-sized particles have many drawbacks such as nontargeting of specific organs, tissues and cells as well as instability, poor aqueous solubility and low bioavailability in human body [7]. Therefore, it is necessary to decrease the size of encapsulated material to sub-micron (0.10–10 μm) and nano size (<0.10 μm).

Due to the increasing prevalence rate of chronic diseases, the emerging challenges in delivering functional compounds to target tissues, organs and cells, as well as instability, poor aqueous solubility and bioavailability, and low release and absorption in vivo could not be overcome by microencapsulation techniques [7]. Recent developments in the field of nanotechnology have provided some excellent means to reduce particle size through top-down (high energy method) or bottom up (self-assembly) processes [29]. Such reduction in particle size has been shown to enhance the stability, targeting ability, bioavailability and release properties [30]. Most importantly, the reduction in particle size enables penetration into deeper portions of cells or tissues resulting in high bioavailability [31]. In the following sections, we have reviewed the research articles published within the last five years on nanoencapsulation of various carotenoid compounds by using different preparation techniques. These studies demonstrated the impact of nanoencapsulation to improve physicochemical property, bioavailability, controlled release and bioactivity. Table 1 and Table 2 summarize various nanosystems used for encapsulation of carotenoids and highlight their advantages as well as disadvantages, respectively.

## 4. Preparation, Physicochemical Characterization, Stability Evaluation and Biological Activity

### 4.1. Nanoemulsion

**Figure 3:**
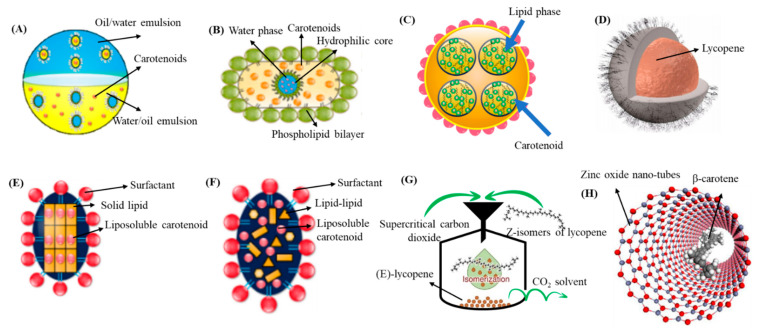
Schematic representation of different nanocarriers for encapsulation of carotenoids. Nanoemulsion (**A**), nanoliposomes (**B**), nanohydrogels (**C**), polymeric/biopolymeric (**D**), solid lipid NPs (**E**), nanostructured lipid carriers (**F**), supercritical fluid-based NPs (**G**) and metal/metal oxide-based NPs (**H**). NPs = nanoparticles. Adapted with permission from Kodama, et al. [75], Monteiro, et al. [76], Rehman, et al. [9], and Vasconcelos, et al. [45].

NEs represent an effective delivery system used for enhancement of physicochemical stability, water dispersibility and bioavailability. They are kinetically stable colloidal systems with small droplet size and improved functional properties [69]. The most commonly used NEs are oil-in-water (o/w) and water-in-oil (w/o) types although multiple emulsion types (o/w/o and w/o/w) are also occasionally used. The preparation methods are classified as high energy and low energy processes with the former involving the use of high energy to break down oil droplets, while the latter generate nanosized oil droplets through mixing of oil, water and surfactant [70]. Among the low energy methods, phase inversion and spontaneous emulsification are the most commonly used ones, whereas mechanical devices such as ultrasonicators, high-pressure homogenizers and microfluidizers are often employed in high energy methods [71]. Nevertheless, some other methods such as direct membrane and premix membrane emulsification methods are also recently used because of less energy consumption and ability to effectively control particle size distribution [72]. By adopting these preparation techniques, NEs with the particle size ≤ 200 nm can be prepared [72,73,74]. Figure 3A shows a schematic representation of o/w and w/o emulsion system [9]. Some NEs developed recently for encapsulation of carotenoids are reviewed in the following section.

**Figure 4:**
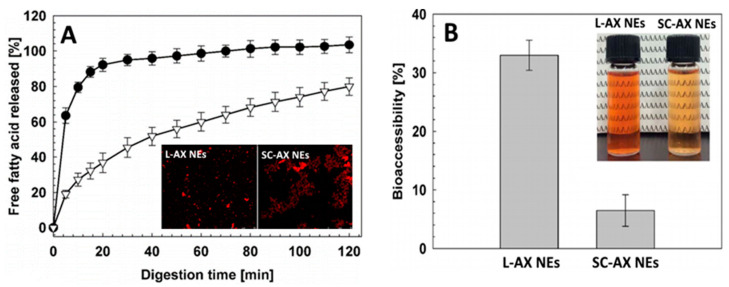
In vitro fatty acid release after digestion of lecithin and sodium caseinate-stabilized astaxanthin NEs in small intestine digestion model. In vitro fatty acid release after digestion of lecithin and sodium caseinate-stabilized astaxanthin nanoemulsions in small intestine digestion model along with an inset showing their respective confocal microscopic images (**A**) and their bioaccessiblity along with the photographs showing the appearance of NEs (**B**). The results were expressed as mean ± standard deviation of ≥ 3 independent replicates and error bars represent the standard deviation from the mean values. L-AX NEs = lecithin-stabilized astaxanthin nanoemulsions and SC-AX NEs = sodium caseinate-stabilized astaxanthin nanoemulsions. Adapted with permission from Khalid, et al. [77].

For encapsulation of a commercial astaxanthin enriched extract (Zanthin^®^), Khalid, et al. [77] developed a o/w NEs by mixing 10% of an organic phase containing 4% astaxanthin extract in soybean oil with an aqueous phase containing 2% emulsifier lecithin or sodium caseinate and 0.02% sodium azide, followed by homogenization in a rotor-stator homogenizer at 10,000 rpm for 5 min and high-pressure homogenization at 100 MPa for 4 cycles. The astaxanthin NEs with a particle size at 115 nm (lecithin-stabilized) or 163 nm (sodium caseinate-stabilized) containing astaxanthin level at 400 μg/mL was shown to possess physical stability during storage for 30 days at 25 °C and chemical stability of >70% following heat treatment at 20–120 °C. Comparatively, the lecithin-stabilized astaxanthin NEs was stable in a wide range of pH and heating temperatures (60–120 °C), while an increase in particle size from 160 to 400 nm occurred at pH 4 and high temperatures (120 °C) for the sodium caseinate-stabilized astaxanthin NEs. On the contrary, sodium caseinate-stabilized NEs were stable even at high NaCl concentration (500 mM). Moreover, the in vitro free fatty acid release and bioaccessibility of lecithin-stabilized astaxanthin NEs was significantly higher than that of sodium caseinate-stabilized NEs, implying that the type of emulsifier can have a strong impact on the bioaccessibility of NEs (Figure 4A,B).

By adopting the same preparation method, Shu, et al. [78] formulated astaxanthin NEs with ginseng saponin as emulsifier by mixing 5% organic phase containing 2% Zanthin^®^ astaxanthin extract in soybean oil with 95% aqueous phase consisting of 0.08–1.20% ginseng saponin, followed by homogenization in a rotor-stator homogenizer at 8000 rpm for 5 min and high-pressure homogenization at 20–100 MPa for 4 cycles. The astaxanthin NPs with a mean particle size of 125 nm were stable to coalescence during storage for 15 days at 5, 25 and 40 °C, or heating at 30–90 °C for 30 min, with a high level of astaxanthin being retained during storage at low temperatures. However, they became unstable at pH 3–6 and sodium chloride >25 mM.

**Figure 5:**
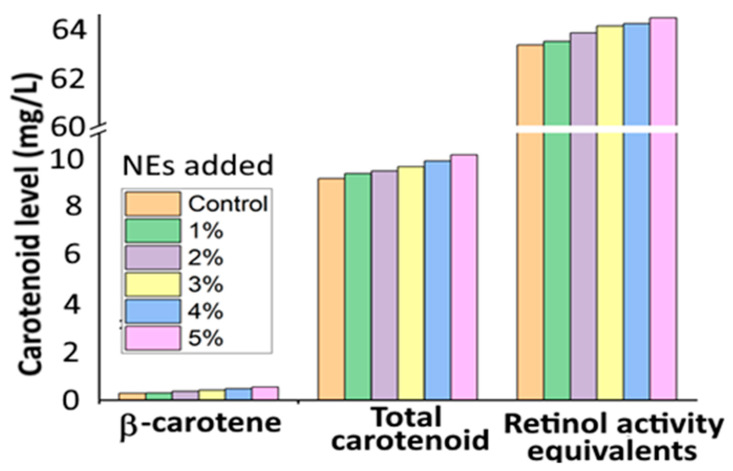
Levels of β-carotene, total carotenoid and retinol activity equivalents in the gastric and pancreatic digests for different levels of carotenoid nanoemulsions (1–5%) prepared from orange peel waste extract. The results were expressed as mean ± standard deviation of ≥ 3 independent replicates. NEs = nanoemulsions. Adapted with permission from Barman, et al. [33].

Several studies have also demonstrated the improved stability, bioaccessibility and photoprotective effect after nanoencapsulation of β-carotene into a suitable NE system. For instance, Barman, et al. [33] developed β-carotene NEs from *Citrus reticulate* (BC-CR NEs) with a particle size at 143.7 nm and zeta potential (ZP) at −38.2 mV by mixing the organic solution (1 g of *C. Reticulate* extract containing 0.28% β-carotene in hexane) with the aqueous phase containing 0.5% polyoxyethylene at a ratio of 1:9, followed by ultrasonic homogenization and vacuum evaporation. The BC-CR NEs were stable at pH 7–10 and heating temperature at 40–70 °C for 30 min, while a high β-carotene retention of 93.7% was shown at 90 °C for 30 min. Also, the BC-CR NEs incorporated into fruit juice at a level of 1–5% showed enhancement of color with a relative bioaccessibility of 32.0–34.7% and retinol activity equivalent of 63.5–64.4% (Figure 5), implying that BC-CR NEs can be an alternative natural food colorant with improved nutraceutical value. 

In another study, Baek, et al. [32] prepared β-carotene-loaded NEs by mixing an appropriate proportion of β-carotene (10 mg), medium chain triglycerides oil (10%), Tween 80 (4.5%), lecithin (4.5%) and deionized water (80%), followed by high-speed homogenization at 5000 rpm for 10 min, and probe sonication for 40 min to obtain β-carotene NEs, which was then mixed with 2% water-soluble chitosan at 1:1 ratio. Compared to uncoated β-carotene NEs, spherically-shaped water-soluble chitosan-β-carotene NEs with a mean particle size at 218 nm and ZP at +40 mV were reported to be stable, as evident by a β-carotene retention of 82% or 77.6% as well as enhancement in thermal stability by 45.1% or 28.6% after 21-day storage at 37 °C or 21-day UV light exposure at room temperature, respectively. Mansur, et al. [34] developed photoprotective NEs using microbial carotenoids (*Microbacterium* sp.) and β-carotene-rich buriti oil (BC-MC/BO NEs) by mixing the organic phase containing 3% buriti oil, 0.2% microbial carotenoids and 0.1% vitamin E with the aqueous phase composed of 12% Tween 80, 3% Span 80 and 2% propylene glycol, followed by homogenization 4 times of 5 min each with 2 min rest in between. A stable BC-MC/BO NEs with a particle size at 142.1 nm and polydispersity index at 0.19 was obtained and shown to have a sun protection factor of 36, suggesting that BC-MC/BO NEs can be used in sunscreen and cosmetic formulations.

To determine the effect of lutein NEs on hepatic steatosis in a guinea pig model, Murillo, et al. [79] prepared o/w lutein NEs by using an appropriate proportion of d-α-tocopheryl polyethylene glycol succinate (8.4%), medium-chain triglyceride (11.2%), water (70.4%) and 10% lutein powder. The lutein NEs with a mean particle size of 254.2 nm, polydispersity index of 0.29 and ZP of −65 mV was obtained and shown to increase the lutein level in plasma and liver by 2- and 1.6-fold, respectively, when compared to lutein powder, indicating a higher bioavailability for the nanoformulated lutein powder. Moreover, a 55% lower hepatic oxidized low-density lipoprotein in both lutein powder and lutein NEs in adipose tissue was shown compared to control, while a 2-fold higher level of low-density lipoprotein and high-density lipoprotein cholesterol was observed in lutein NEs-treated group than that in lutein powder-treated group, indicating that lutein NEs could exert a protective effect against hepatic steatosis, but elevate the cholesterol level in adipose tissue.

In a later study, Hsu, et al. [80] prepared NEs from zeaxanthin -rich carotenoid extract from *Lycium barbarum L.* (ZX-CE-LB NEs) for evaluating their inhibition mechanism towards colon cancer cell HT-29. By homogenizing an appropriate proportion of dried *L. barbarum* carotenoid extract (30 mL) with Capryol^TM^90 (0.2 mL), Transcutol^®^HP (0.4 mL), Tween 80 (1 mL) and deionized water (8.4 mL) by ultrasonication, ZX-CE-LB NEs with a mean particle size of 16 nm, ZP of −68 mV and EE of 98% were formulated with a high stability over a 90-day storage period at 25 °C. Also, a higher in vitro release rate was shown at pH 5.2 than at pH 7.4, suggesting that a rapid carotenoid release can occur under the acidic tumor environment (pH 4.5–6.5). Moreover, the ZX-CE-LB NEs was efficient in inhibiting the growth of HT-29 cells with a half maximal inhibitory concentration (IC_50_) of 4.5 μg/mL by arresting cell cycle at G_2_/M phase through up-regulating p53 and p21 expressions as well as down-regulating CDK2, CDK1, cyclin A and cyclin B expressions.

With an aim to enhance the antioxidant activity and bioaccessiblity of lycopene-enriched tomato extract, Ha, et al. [81] prepared lycopene NEs using the emulsification-evaporation method by mixing an organic phase containing 6% lycopene in ethyl acetate and 0.01% butylated hydroxytoluene with an aqueous phase containing 0.5% Tween 20, followed by homogenizing with a shear homogenizer at 5000 rpm for 5 min and passing through high-pressure homogenization (60–140 MPa, 1–3 cycles). The particle size of lycopene NEs ranged from 96–282 nm, ZP from −33 to −42 mV and EE from 51.6 to 65.3%, while a decline in EE was shown following a rise in homogenization cycle from 1–3. Interestingly, the lycopene NEs with a particle size ranging from 100–200 nm was shown to exhibit high antioxidant activity based on the 2,2-diphenyl-1-picrylhydrazyl (DPPH) assay (IC_50_, 190.8 g lycopene/kg DPPH) and Trolox equivalent antioxidant capacity (TEAC) assay (235.3 μM TEAC) compared to lycopene NEs with a particle size <100 nm (156 g lycopene/kg DPPH and 261 μM TEAC). The in vitro bioaccessibility of tomato extract, lycopene NEs with size >100 nm and <100 nm was 0.01, 0.53 and 0.77, respectively, suggesting that a smaller particle size of lycopene NEs could enhance its bioaccessibility. 

In a later study, Li, et al. [38] developed lycopene NEs by mixing an aqueous solution of octenyl succinic anhydride-modified starch powder (30%) with an organic phase containing 0.1–0.5% lycopene dispersed in medium-chain triacylglycerol at a ratio of 1:9, followed by emulsification at 18,000 rpm for 4 min and high-pressure homogenization at 110 MPa for three cycles. Spherically-shaped lycopene NEs with a particle size ranging from 145.1 to 162.0 nm, ZP from −19.7 to −20.8 mV was reported to possess high physical stability as measured by Turbiscan AGS stability analysis. Also, both Raman and NMR analyses revealed the presence of lycopene in the hydrophobic core of NE at low lycopene level (0.1%), while at a high lycopene level (0.5%), lycopene was found in both the core and interface of NE, strengthening the lateral packing of octenyl succinic anhydride molecules in NEs’ interfacial region. 

In another study, NEs containing astaxanthin or lycopene with a particle size at <200 nm and ZP from −30 to −45 mV were prepared by mixing 99% of the aqueous phase containing 0.5% Tween 20 and 1% organic phase containing 2 g/L astaxanthin or 1 g/L lycopene dispersed in 0.5% linseed oil, followed by homogenization at 5000 rpm for 10 min and high-pressure homogenization at 5–100 MPa for 1–10 cycles [37]. The incorporation of 6-hydroxy−2,5,7,8-tetramethylchroman-2-carboxylic acid (Trolox) alone or in combination with butylated hydroxytoluene was shown to increase the oxidative stability of both NEs. An in vitro study on mouth, gastric and intestinal-based digestion models revealed a faster carotenoid release and lower free fatty acid content for NEs prepared with high-pressure homogenization at 100 MPa, while a partial carotenoid absorption (66%) and >70% bioaccessibility for different particle size of carotenoid NEs was obtained at different homogenization pressure.

Some other studies have also reported the development of carotenoid based NEs [82,83,84]. Comparatively, NEs are among the nanocarotenoid carriers most frequently used in pharmaceutical, food and chemical industries. Although NEs offer several advantages, the future studies should focus on developing NE formulations with long-term stability, enhanced bioavailability and biological activity in vivo.

### 4.2. Nanoliposomes

NLs are the most commonly investigated colloidal delivery system with spherical vesicles, consisting of a phospholipid bilayer with a hydrophilic core as illustrated in a schematic Figure 3B [3,9]. Owing to their biocompatibility and ability to encapsulate both hydrophobic and hydrophilic compounds, NLs have been the choice for encapsulating a wide variety of bioactive compounds. Based on the lipid bilayer, NLs are classified as unilamellar and multilamellar with the particle size ranging from <100–1000 nm and 500–5000 nm, respectively. Unilamellar consists of single lipid bilayer, while a large number of lipid bilayers constitute a multilamellar liposome [85]. The major advantages of NLs are increased stability, reduced toxicity of encapsulated compounds and improved bioavailability. However, they have several drawbacks including short shelf-life, low EE, microbial spoilage and difficulty in controlling the liposome size [5,85]. Accordingly, the NLs can be prepared either by application of mechanical energy such as sonication, high-pressure homogenization, microfluidization and membrane homogenization, or by non-mechanical methods such as thin film hydration, solvent injection, detergent removal and reverse phase evaporation [5,85]. Recently, many more advanced techniques have been developed including the freeze-drying double method, dense-gas technique, cross-flow filtration technology, membrane-contractor technology, dual-asymmetric centrifugation and supercritical-fluid technology [9]. Therefore, the selection of an appropriate method for preparation of NLs is important. 

De Freitas Zômpero, et al. [86] prepared β-carotene-loaded NLs by an ethanol injection method for further preparation of β-carotene NFs by an electrospinning process. Initially, NLs were prepared by dispersing 0.5% β-carotene in phospholipids, followed by injecting at 30 mL/min into a jacketed reactor containing 100 mL of water and stirring for 5 min at 60 °C. Then, the β-carotene NFs were obtained by mixing the β-carotene NLs with PVA/polyethylene oxide solutions at a ratio of 2.5–7.5% to obtain ultrathin NFs with enhanced β-carotene stability and UV photostability. More recently, Hassane Hamadou, et al. [52] developed β-carotene loaded NLs by a thin-film evaporation method by mixing β-carotene in ethanol with marine phospholipids or egg phospholipids at a ratio of 5:1 (*v/w*), followed by vacuum evaporation at 55 °C, hydration with phosphate buffer (0.01 M, pH 7.4) and sonication at 240 W for 9 min. The particle size of β-carotene NLs ranging from 162.9–365.8 nm, ZP from 42.6–64.5 mV and EE from 96.5–99% were obtained with β-carotene NLs prepared from marine phospholipids (42.9%) showing a higher stability (4 days at 4 °C) and inhibition against lipid peroxidation than that from egg phospholipids (10.3%).

To enhance the stability of astaxanthin, Pan, et al. [53] prepared astaxanthin NPs by a thin-film ultrasound method with 25 mL of 0.05 M phosphate buffer (pH 7.4), soybean phosphatidylcholine to cholesterol ratio at 5:1, and ultrasonication time at 4 min. The astaxanthin NPs were obtained with a mean particle size at 80.6 nm, polydispersity index at 0.2, ZP at −31.8 mV and EE at 97.7%, however, it showed a decrease in EE to 82.2% and 61.3%, respectively, upon storage at 4 °C and 25 °C for 15 days, revealing a more efficient retention of encapsulated carotenoid only at low storage temperature. The astaxanthin loss during storage may be caused by hydrolysis and oxidation of phospholipids, resulting in alteration of the NL bilayer structure. In addition, compared to free astaxanthin, the 1% astaxanthin-loaded NLs showed a higher antioxidant activity in terms of DPPH radical scavenging activity (75.1%) and lipid peroxidation inhibition (67%). In a follow-up study, an enhancement of both thermal stability and water dispersibility was shown after encapsulation of astaxanthin into NLs. Also, a decreased membrane-fluidity and increased micro-polarity was observed for astaxanthin NLs based on the steady-state fluorescence measurement [54]. This outcome suggests that astaxanthin encapsulation can be applied to modulate the structural properties of the NL membrane. 

More recently, shrimp oil, a rich source of *n*-3 fatty acids and astaxanthin, was used for preparation of astaxanthin encapsulated NLs by both ultrasonication and microfluidization methods for evaluating the oxidative stability [87]. The shrimp oil-NLs were prepared by mixing preheated shrimp oil (5 mL) and lecithin (100 mL), followed by stirring at 30 °C, hydrating with 100 mL of deionized water and 2% of glycerol, homogenizing for 10 min and ultrasonicating at 80% amplitude for 10 min at 25 °C. Then, the hydrated mixture (200 mL) was passed through a high-pressure microfluidizer (10 cycles) at 7000 psi and evaporated at 30 °C. The ultrasonication-based shrimp oil-NLs (particle size 104.8 nm and EE 93.6%) were shown to exhibit higher thermal stability at 220 °C than the microfluidization-based shrimp oil (particle size 512.8 nm and EE 75.2%), while their respective EE remained unchanged upon storage at 30 °C for 8 weeks, implying a higher oxidative stability for the ultrasonication-based encapsulated shrimp oil.

**Figure 6:**
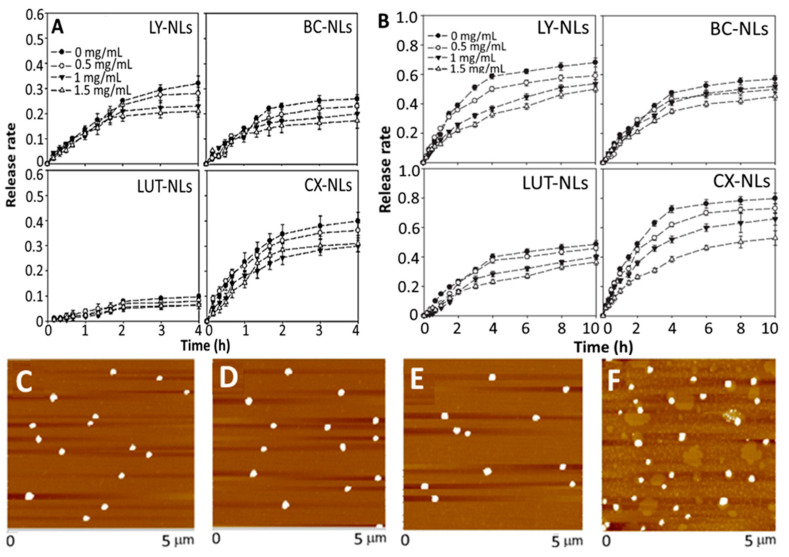
In vitro release of lycopene, β-carotene, lutein and canthaxanthin from respective carotenoid nanoliposomes and chitosan-coated carotenoid nanoliposomes (0.5–1.5 mg/mL chitosan) in simulated gastric fluid (**A**), intestinal fluid (**B**) along with the atomic force microscopic images of lycopene nanoliposomes (**C**), β-carotene nanoliposomes (**D**), lutein nanoliposomes (**E**) and canthaxanthin nanoliposomes (**F**). The results were expressed as mean ± standard deviation of ≥ 3 independent replicates and error bars represent the standard deviation from mean values. NLs = nanoliposomes, LY = lycopene, BC = β-carotene, LUT = lutein and CX = canthaxanthin. Adapted with permission from Tan, et al. [51] and Xia, et al. [88].

Polymers such as chitosan and poly-L-lysine are also employed for preparation of carotenoid NLs to achieve enhanced stability as well as in vitro release and absorption. For example, Tan, et al. [51] prepared phospholipid and chitosan-NLs for encapsulation of lycopene, β-carotene, lutein and canthaxanthin by a thin-film evaporation method. A mixture containing carotenoid and mixed lipid (egg yolk phospholipid + Tween 80; 99:1, *w/w*) was dried by vacuum evaporation at 55 °C, followed by hydration with 40 mL of phosphate/acetic acid/sodium chloride (pH 4.0), vortexing for 60 min and probe sonication for 10 min at 240 W. For chitosan coating, the chitosan was dissolved in the same buffer solution and added dropwise into carotenoid-NLs for 30 min with stirring at 1000 rpm. Then, the chitosan-coated carotenoid NLs was obtained with particle size at 70–100 nm, ZP at −5.3 mV and EE at 75%, while a high stability upon heating at 37 °C for 6 h, 65 °C for 30 min and 90 °C for 30 s, was shown. Also, it was effective against centrifugal sedimentation and gastrointestinal stress (0.06–0.31% release over 4 h) with the release rate in stimulated gastric and intestinal fluids being shown in Figure 6A,B. Most importantly, the encapsulated carotenoids were retained to a different degree depending on molecular structure, with β-carotene and lutein being more efficiently protected than lycopene and canthaxanthin. In an earlier study, the same authors also reported that the incorporation of four carotenoids (lycopene, β-carotene, lutein and canthaxanthin) into NLs could significantly change morphology and particle size of NLs from 20 to 425 nm and their AFM images are shown in Figure 6C–F [88]. Moreover, the dynamics, structure (difference in choline group conformation of polar head and acyl chain) and hydrophobicity (polar headgroup region and hydrophobic core) of NLs membrane were modulated depending on the molecular structure and carotenoid concentration, which in turn affected the NLs stability in terms of particle aggregation and flocculation. Obviously, the practical application of NLs poses serious challenges as NLs are susceptible to aggregation or particle fusion leading to leakage of entrapped compounds. This phenomenon can be influenced by the structure and conformation of entrapped functional compounds. More specifically, lutein and β-carotene are capable of lowering the membrane fluidity and bilayer hydrophobicity of NLs more effectively than lycopene and canthaxanthin, both of which are more prone to aggregation than the former ones [88]. Also, the entrapped carotenoids can significantly modify the physical properties of NL’s membrane in a concentration-dependent manner. This important finding provides a basis for designing NLs for carotenoid encapsulation and application in functional foods/drugs developments.

**Figure 7:**
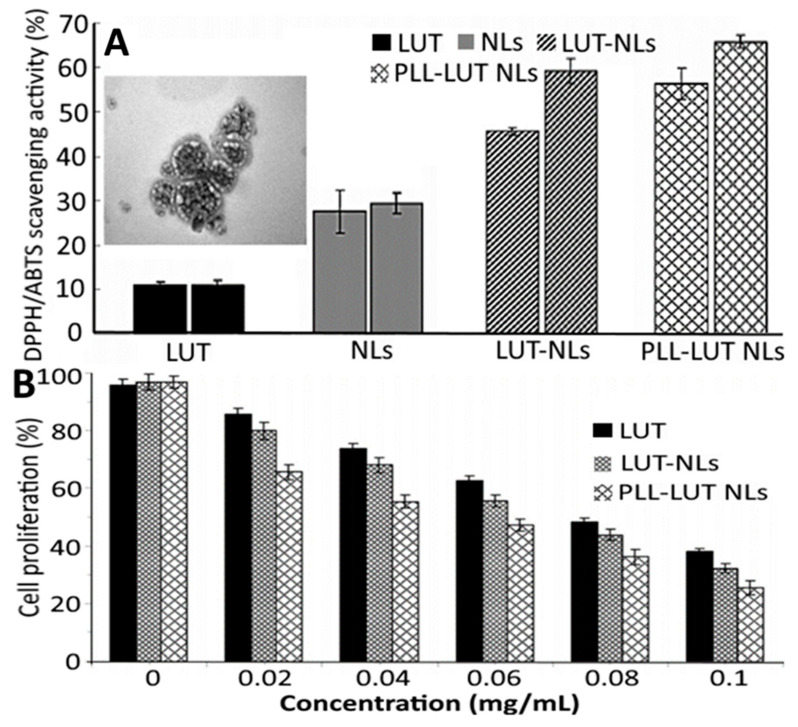
Antioxidant activity in terms of 2,2-diphenyl-1-picrylhydrazyl and 2,2′-azino-bis (3-ethylbenzothiazoline-6-sulfonic acid) scavenging activity for blank nanoliposomes, free lutein, lutein nanoliposomes and poly-L-lysine coated lutein nanoliposomes along with an inset showing the transmission electron microscopic image of poly-L-lysine coated lutein nanoliposomes (**A**) as well as their anti-proliferative effects on Caco-2 colon cancer cell (**B**). The results were expressed as mean ± standard deviation ≥ 3 independent replicates and error bar represents the standard deviation from mean values. NLs = nanoliposomes, PLL- poly-L-lysine and LUT = lutein. Adapted with permission from Jiao, et al. [55].

In a later study, Jiao, et al. [55] employed an ethanol injection method for production of poly-L-lysine (PLL) decorated lutein-NLs by mixing lutein, cholesterol, Tween 80 and lecithin in ethanol at an optimized ratio of 1:10:40:10, followed by adding 0.05 M phosphate-buffer solution (pH 6) with stirring for 30 min at 50 °C, evaporating at 50 °C and hydrating under reduced pressure. To enhance the bioavailability, the lutein NLs were further decorated with PLL (0.04–0.08%) in phosphate-buffer solution by incubating at 50 °C for 120 min and cooling at room temperature. Depending on the PLL level, the particle size, ZP and EE of lutein NLs varied respectively from 264.8 to 367.1 nm, −27.9 to −34.3 mV and 91.8 to 92.9%, with the highest EE being shown for lutein NLs with 0.06% PLL coating. Compared to uncoated lutein NLs (43.3% and 53.8%), the PLL-coated lutein NLs showed a higher release in both simulated gastric and intestinal fluids (51.3% and 70.3%). Also, the lutein NLs exhibited a higher antioxidant activity compared to free lutein and blank NLs, with PLL coating showing only a slightly higher activity (Figure 7A). Likewise, the PLL-coated lutein NLs showed a higher inhibitory effect towards Caco-2 colon carcinoma cells (84%) than uncoated lutein NLs (68%) and free lutein (60%), probably because PLL could increase Caco-2 uptake by enhancing the permeability through the cell membrane (Figure 7B). 

Overall, the above-mentioned studies highlighted the application of NLs as an efficient carotenoid carrier to improve the solubility, stability and antioxidant activity of encapsulated carotenoids. Nevertheless, some more studies are needed for encapsulation of carotenoids in bilosomes (non-ionic amphiphiles unified with bile salts) and niosomes (non-ionic surfactants) for possible application in functional food or drug industry.

### 4.3. Polymeric/Biopolymeric Based Nanoparticles

Polymeric/biopolymeric-based NPs have received considerable attention in nanoencapsulation of carotenoids due to their enhanced biocompatibility, bioaccessibility and controlled release of carotenoids during digestion [3]. More importantly, they enable longer circulation of bioactive compounds in blood and higher accumulation in tissues, cells and organs [89]. The particle size of polymeric NPs ranges from 10–1000 nm and are composed of either natural polymer such as chitosan, gelatin, casein, κ-carrageenan, sodium alginate, albumin and heparin or synthetic polymers such as polyethylene glycol, poly-L-lactic acid, polycaprolactone, poly (alkyl cyanoacrylate) and *N*-(2-hydroxypropyl)-methacrylamide [90,91]. Among various biopolymers used for preparation of polymeric NPs, chitosan, a cationic natural polysaccharide, is the most commonly used natural polymer owing to its cross-linking capacity and ability to provide sustained release of encapsulated bioactive compounds [92]. Most importantly, it is classified as Generally Recognized as Safe (GRAS) by the United States Food and Drug Administration (USFDA) and thus can be used either alone or in combination with other polymers for preparation of carotenoid NPs with enhanced physicochemical and biological characteristics [92]. In practice, chitosan can be used in combination with alginate [93], poly-γ-glutamic acid [94] and poly(lactide-co-glycolide) (PLGA) [95] for preparation of polymeric NPs. 

Besides NEs, NLs and NPs, the unique nanostructures involving synthetic polymers of biopolymers such as nanogel/nanohydrogel, NFs, micelle, nanotube and NCs were also prepared. A schematic of nanohydrogel used in the food and cosmetics industries is shown in Figure 3C [9]. Depending on the type of bioactive compound and polymeric system, polymers are directly incorporated or through polymerization of monomers for preparation of polymeric NPs. For instance, nanoprecipitation, solvent evaporation, solvent diffusion and emulsification are frequently used methods with pre-synthesized polymers as raw material [96]. Preparation of polymeric NPs by a preformed polymer method usually involves injection of an organic phase containing polymer into an aqueous phase containing solvent and bioactive compound [5]. Several advantages of using polymeric NPs over the other types of NPs include simple preparation, nontoxic nature, targeted drug delivery, high stability, biocompatibility as well as increased stability of biological compounds [97]. 

#### 4.3.1. Nanoparticles

**Figure 8:**
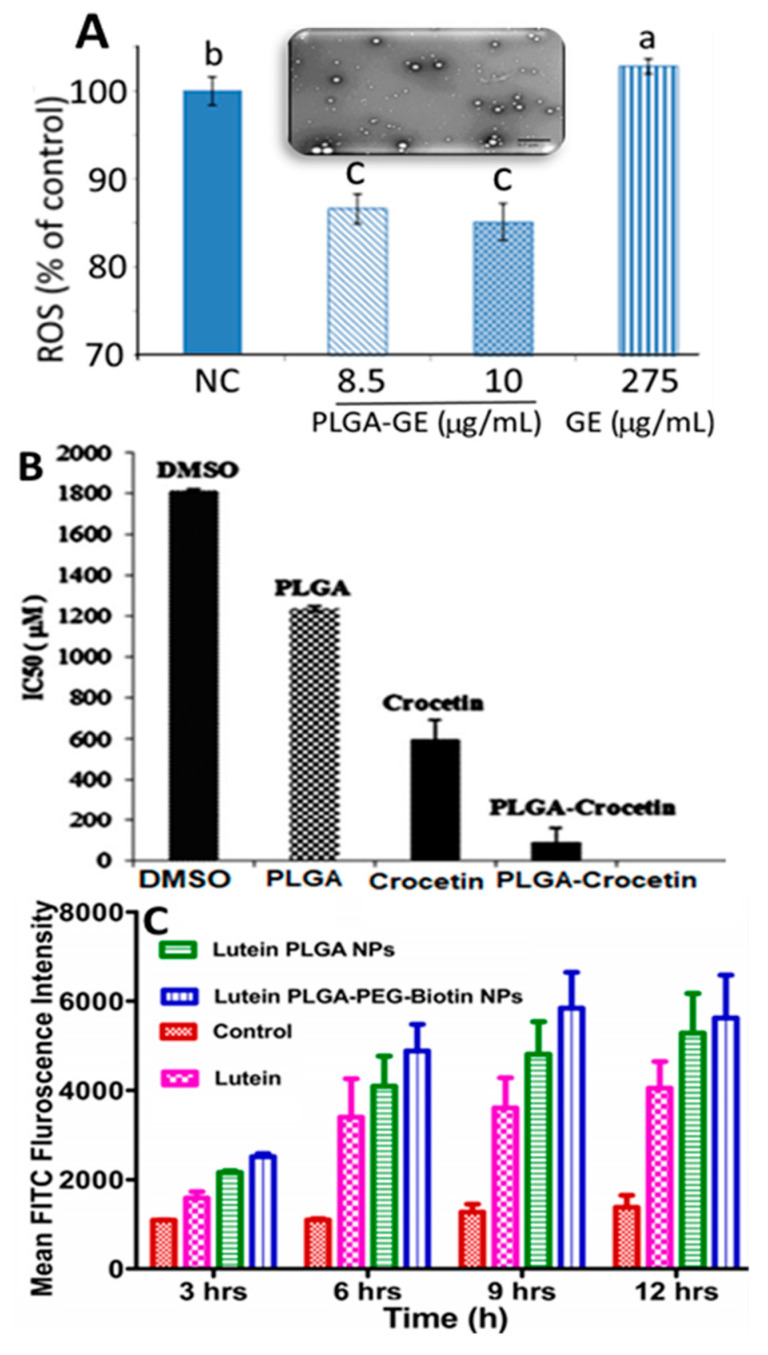
Poly(lactic-co-glycolic acid)-based polymeric nanoparticles for nanoencapsulation of carotenoid-rich *Guabiroba* fruit extract (**A**), crocetin (**B**) and lutein (**C**). Panel A illustrating generation of reactive oxygen species in HT-29 colon cancer cells treated with poly(lactic-co-glycolic acid)-based *Guabiroba* fruit extract nanoparticles along with an inset showing their transmission electron microscopic image, while panel B depicting half-maximal inhibitory concentration value for cytotoxicity effects of dimethyl sulfoxide, free crocetin and poly(lactic-co-glycolic acid)-crocetin nanoparticles on human breast cancer cells MCF-7 (**B**) as well as panel C showing fluorescence activated cell sorting analysis of control, free lutein, lutein-poly(lactic-co-glycolic acid) nanoparticles and lutein-loaded poly(lactic-co-glycolic acid)-polyethylene glycol-biotin nanoparticles after treatment with human retinal pigment epithelial cells ARPE-19 at different time periods (3–12 h). The results were expressed as mean ± standard deviation of ≥ 3 independent replicates and error bars represent the standard deviation from mean values, while different lowercase letters above each bar in (**A**) represent significantly different values. DMSO = dimethyl sulfoxide, PLGA = poly (lactic-co-glycolic acid), PEG-polyethylene glycol, FACS = fluorescence activated cell sorting, FITC = fluorescein isothiocyanate, GE = *Guabiroba* fruit extract, ROS = reactive oxygen species and IC_50_ = half maximal inhibitory concentration. Adapted with permission from Pereira, et al. [40], Hafezi Ghahestani, et al. [49], and Bolla, et al. [47].

Encapsulation of both carotenoid extract and standard were reported to improve stability and bioavailability for enhanced biological activity. Like chitosan, PCL and PLGA are the two other polymers most often used for encapsulation of carotenoids. For example Pereira, et al. [40] prepared *Guabiroba* extract-PLGA NPs from a 50:50 ratio of PLGA:*Guabiroba* extract by an emulsion-evaporation method and reported a particle size of 153 nm, EE of 83.7% and in vitro release of 92% after 1 h. They were shown to be effective in inhibiting growth of *Listeria innocua* in a concentration range of 954–1200 mg/mL. *Guabiroba* extract-PLGA NPs also exerted a 6–10-fold higher DPPH scavenging activity compared to free *Guabiroba* extract, whereas both treatments scavenged the ABTS (2,2′-Azino-bis(3-ethylbenzthiazoline-6-sulfonic acid) to the same extent. Compared to *Guabiroba* extract (275 µg/mL), the *Guabiroba* extract-PLGA NPs could reduce reactive oxygen species generated in colon cancer cells HT-29 at a much lower dose (8.5 and 10 µg/mL *Guabiroba* extract in PLGA NPs) (Figure 8A), suggesting that *Guabiroba* extract-PLGA NPs could possess antioxidant and antibacterial properties. Similarly, by employing a solvent evaporation method, Hafezi Ghahestani, et al. [49] encapsulated crocetin using a PLGA-polyvinyl alcohol (PVA) polymer to obtain PLGA-PVA-crocetin NPs with a particle size ranging from 288 to 584 nm and EE from 59.6 to 97.2% and demonstrated an in vitro release of 44.5 and 96% in phosphate buffered saline (pH 7.4) after incubation for 24 h and 4 days, respectively, indicating that the PLGA polymer can facilitate sustained release of crocetin. Moreover, compared to free crocetin (589.7 µM), the PLGA crocetin NPs showed a lower IC_50_ value (84.7 µM) in inhibiting human breast cancer cell MCF-7 (Figure 8B). Also, in a recent study Bolla, et al. [47] used biotin-based PLGA-polyethylene glycol (PEG) for encapsulation of lutein to develop PLGA-PEG-lutein NPs (particle size, <250 nm; ZP, −27.3 mV; EE, 74.6%) and showed a sustained 100% in vitro release after 24 h. Furthermore, they showed a higher lutein intake by retinal pigment epithelial cell ARPE-19 from PLGA-PEG-lutein NPs than from both free lutein and PLGA- lutein NPs (Figure 8C), demonstrating that a combination of polymers could possess a greater potential in treating age-related macular degeneration.

For poly-ε-caprolactone (PCL) polymer-based carotenoid NPs, Dos Santos, et al. [46] developed PCL-lycopene NPs by interfacial deposition through mixing an organic phase containing 200 mg of PCL, 320 μL of caprylic/capric triglyceride, 76 mg of sorbitan monostearate and 93.9% of lycopene in acetone/ethanol (8:1) with an aqueous phase containing Tween 80 (154 mg), followed by stirring at 40 °C (10 min) and evaporating under reduced pressure to obtain PCL-lycopene NPs containing 85 μg/mL lycopene. The PCL-lycopene NPs (particle size, 193 nm; ZP, −11.5 mV) were shown to be stable over the first 14-day storage period with no particle size change and 50% lycopene retention at room temperature, while a change in particle size (>193 nm) was observed during storage from 14 to 30 days. However, no biological activity was determined for the PCL-lycopene based NPs. In a recent study dealing with preparation of PCL-lycopene NPs from red guava extract (*Psidium guajava* L.), Vasconcelos, et al. [45] demonstrated their enhanced stability and inhibitory effects on breast cancer cell MCF-7 as well as alleviation of LPS-induced oxidative stress in microglial cells HMC3 by using a time-lapse microscopy. More elaborately, an organic phase containing PCL in acetone, sorbitan monostearate, coconut oil and lycopene extract in ethanol at a ratio of 60.81:14.77:0.10:1.74 was mixed with 190 mL of an aqueous phase containing Tween 80 (150 μL), followed by stirring at 40 °C for 10 min and evaporating under reduced pressure to 10 mL at 37 °C. The spherically-shaped PCL-lycopene NPs (particle size, 200 nm; ZP, −26.2 mV; EE, 96.3%) was highly stable even after storage for 7 months at 5 °C and effective in inhibiting the growth of MCF-7 cells by 61.5 and 56%, respectively, after 24 and 72 h incubation. In addition, PCL-lycopene NPs were effective in mitigating LPS-induced NF-κB activation and reactive oxygen species production in microglial cells. 

In another study, Figueiredo-Junior, et al. [98] developed PCL-based bixin NPs by interfacial deposition method and evaluated their ability in alleviating acute lung inflammation in mice induced by cigarette smoke exposure. Through mixing the annatto seed-derived bixin in PCL-acetone solution at a ratio of 1:10 with an aqueous phase containing 1% poloxamer 188 at 40 °C, the PCL-bixin NPs were obtained with a mean particle size at 23.7 nm, ZP at −21.7 mV, EE at 48.5% and bixin loading at 24.2 mg/g. Following treatment of cigarette smoke-exposed mice with 100 μL of 12% and 18% PCL-bixin NPs, the levels of superoxide anion and total bronchoalveolar lavage leukocyte were reduced by 53.8% and 35.7%, respectively, which can be attributed to the antioxidant and anti-chemotactic activity of bixin.

Several other studies have also compared different food-based polymers used for preparation of carotenoid NPs for evaluation of stability, antioxidant activity and bioavailability. Yi, et al. [42] prepared β-carotene NPs using three different food-based proteins including sodium caseinate (SC-BC NPs), whey protein isolate (WPI-BC NPs) and soybean protein isolate (SPI-BC NPs) by homogenization-evaporation method using ethyl acetate as solvent, with the mean particle size, ZP and EE ranging from 77.8 to 371.8 nm, −37.8 to −29.9 mV and 98.7 to 99.1%, respectively. The in vitro release of WPI-BC NPs was slow with pepsin at pH 2, but high with trypsin at pH 7, suggesting that WPI may be an efficient protein delivery vehicle to carry β-carotene from intestine to tissue. Compared to free β-carotene (16.7 and 17.6%), both DPPH and hydroxyl scavenging activity for SC-BC NPs (69.6 and 49.9%), WPI-BC NPs (72.8 and 46.5%) and SPI-BC NPs (66.8 and 42.4%) were higher, implying an enhanced antioxidant activity after β-carotene incorporation into polymeric NPs. Also, the cellular antioxidant activity (EC_50_) of all the three protein-based β-carotene NPs (SC-BC, WPI-BC and SPI-BC = 14.4, 13 and 17.1 µg/mL, respectively) were higher than that of free β-carotene (24.8 µg/mL). Likewise, some other studies have also demonstrated the importance of preparing polymeric NPs for β-carotene encapsulation [99,100]. In a later study, the yellow passion fruit albedo flour pectin-based carotenoid-rich NPs prepared from a microalga *Spirulina* extract by solvent displacement method (particle size, 84.4 nm; ZP, −41.4 to −43.6 mV; EE, 97%) were shown to possess high physicochemical stability over a 60-day storage period at 4 °C in terms of particle size, DPPH scavenging activity (60%) and carotenoid retention in microalgae extract (63.3%) and albedo flour (58.8%) [41].

**Figure 9:**
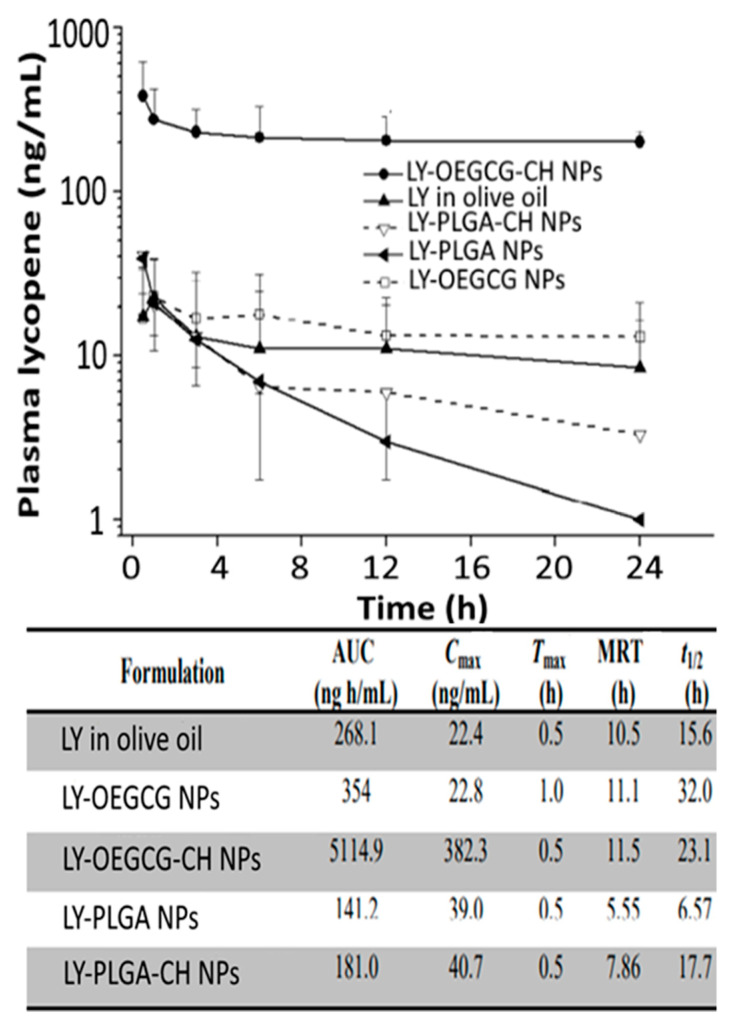
Time profiles on mouse plasma lycopene levels after treatment with lycopene in olive oil, lycopene-poly(lactic-co-glycolic acid) nanoparticles, lycopene-oligomerized epigallocatechin gallate nanoparticles, lycopene-poly(lactic-co-glycolic acid)-chitosan nanoparticles and lycopene-LY- oligomerized epigallocatechin gallate-chitosan nanoparticles along with a footnote table showing their corresponding pharmacokinetic parameters area under curve (AUC), maximum plasma lycopene concentration (C_max_), time taken to reach C_max_ (T_max_), mean residence time (MRT) and time required for the concentration of the drug to reach half of its original value (t_1/2_). The results were expressed as mean ± standard deviation of ≥3 independent replicates and error bars represent the standard deviation from mean values. LY = lycopene, OEGCG = oligomerized epigallocatechin gallate nanoparticles, CH = chitosan and PLGA = poly (lactic-co-glycolic acid). Adapted with permission from Li, et al. [44].

Likewise, chitosan, PLGA, chitosan-PLGA, green tea-based oligomerized (-)epigallocatechin-3-O-gallate (OEGCG) and chitosan-OEGCG were compared by Li, et al. [44] for encapsulation of lycopene by nanoprecipitation method and evaluation of in vitro release in simulated gastric/intestinal fluids and pharmacokinetic analysis in mice. The chitosan-OEGCG-lycopene NPs with a mean particle size of 152 nm, ZP of 58.3 mV and EE of 89% showed a slow release (5%) of lycopene in simulated gastric fluid at pH 2 and faster release (12%) in simulated intestinal fluid (Figure 3D). Compared to lycopene in olive oil, PLGA-lycopene NPs, chitosan-PLGA-lycopene NPs and OEGCG-lycopene NPs, the chitosan-OEGCG-lycopene NPs administered orally into mice at 10 mg/kg body weight showed a higher level of lycopene in mice serum based on the pharmacokinetic parameters AUC (area under the curve) at 5114.9 ng/mL, C_max_ at 382.3 ng/mL, T_max_ at 0.5 h, mean residence time at 11.5 h and t_1/2_ at 23.1 h (Figure 9), implying that a combination of the polymer chitosan with OEGCG can be an efficient strategy for enhancement of lycopene stability and bioavailability.

#### 4.3.2. Nanofiber, Nanocapsules and Micelles

**Figure 10:**
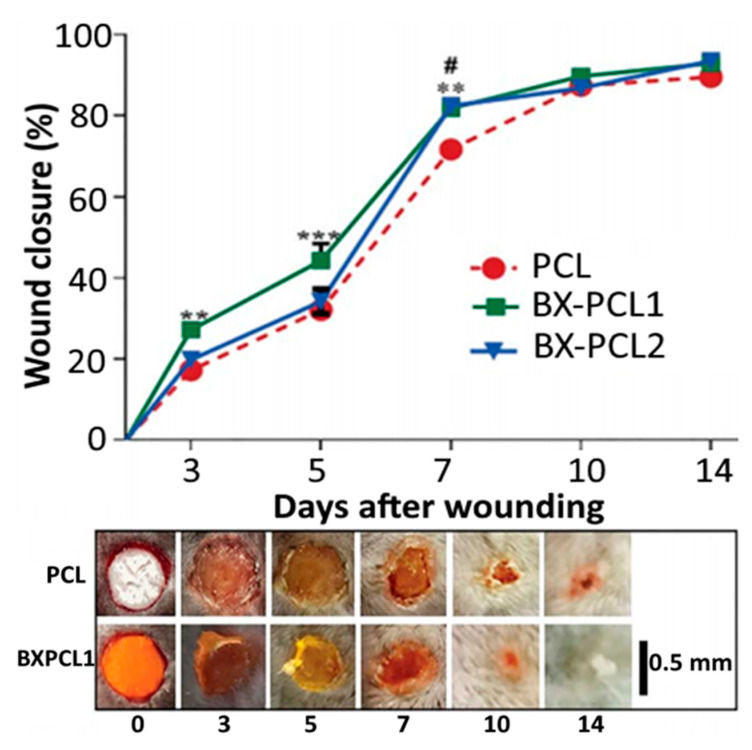
Diabetic wound healing property of bixin-polycaprolactone nanofibers in terms of their wound closure percentage in an excisional skin injury on diabetic mice as a function of time and nanoencapsulated bixin level (2.5 and 12.5% bixin as BX-PCL1 and BX-PCL2) along with their scanning electron microscopic image as well as macroscopic pictures of wound closure. The results were expressed as mean ± standard deviation of ≥ 3 independent replicates and error bars represent the standard deviation from mean values, while **, *** and # symbols represent significantly different values. BX = bixin and PCL = polycaprolactone. Adapted with permission from Pinzón-García, et al. [102].

An apocarotenoid bixin-based NCs using PCL polymer was prepared by Lobato, et al. [101] by interfacial deposition through mixing of an organic phase containing 250 mg of PCL, 400 mL of caprylic/capric triglyceride, 95 mg of Span 60 and 0.41 mg of bixin in acetone/ethanol (8:1, *v/v*) with 130 mL of an aqueous solution containing 195 mg of Tween 80, followed by stirring for 10 min and evaporating under vacuum to 25 mL. Compared to free bixin, the PCL-bixin NPs with a mean particle size of 190 nm, ZP of −14.5 mV and EE of 100% showed a higher stability in an ethanol/water (1:4, *v/v*) model system photosensitized with 150 W filament lamp (36000 lux) at 5–25 °C and heated at 65–95 °C. In another study Pinzón-García, et al. [102] prepared PCL-bixin NFs with diabetic wound healing property by an electrospinning process. The PCL solution in 50:50 dichloromethane/methanol was mixed with 2.5 or 12.5% bixin, followed by injection with a syringe pump (10 mL/h) under high voltage at 20 mV, collecting PCL-bixin NFs on an aluminum foil-wrapped collector and drying overnight at 40 °C. The spherically-shaped PCL-bixin NFs obtained with a particle size of 341–445 nm showed an initial burst in vitro release from 30–40% of bixin in 10 h and a maximum (100%) was attained after 14 days. Also, they could accelerate diabetic wound healing and reduce scar tissue area in an excised mice diabetic wound model (Figure 10). In a later study, Muhoza, et al. [103] nanoencapsulated lutein into glycosylated casein micelles by an ethanol injection method combined with ultra-high pressure homogenization. More elaborately, lutein micelle with a particle size of 118.5 nm and EE of 97.2% was prepared by injecting 2 mL of lutein in ethanol into an aqueous solution of glycosylated casein, followed by stirring for 30 min, evaporating under reduced pressure at 55 °C and homogenizing at 1050 bar at 1st cycle and 1400 bar at 2nd cycle, with lutein being conjugated to the hydrophobic portion of glycosylated casein.

More recently, the glucuronoxylan-based β-carotene NPs/NFs were prepared from *Cydonia oblonga* mucilage extract by dissolving β-carotene extract in corn oil, mixing with an aqueous phase containing 0.3% freeze-dried *Cydonia oblonga* mucilage and sonicating in an ice bath for 2 min [43]. The amorphous β-carotene NPs with an average diameter of 70.5 nm and EE of 97.5% was shown to be thermally stable. Moreover, following a rise in β-carotene level (2.5 to 20%), the viscosity and droplet size increased, but both conductivity and surface tension diminished, accompanied by a transition from NPs into NFs by an electrohydrodynamic process. More recently, Vieira, et al. [104] prepared astaxanthin-rich NCs from *Haematococcus pluvialis* (AX-HP NCs) by solvent displacement method using PLGA polymer and demonstrated their antioxidant activity. The AX-HP-NCs possessing a particle size of 215.4 nm, ZP of −40.8 mV and EE >98% with a total carotenoid content ranging from 106.4 to 156.2 μg/mL exhibited a high antioxidant activity by DPPH assay with an IC_50_ being 3–9 fold higher than that for ascorbic acid, indicating that the encapsulated *H. pluvialis* carotenoids can enhance the antioxidant activity of AX-HP NCs. The authors further added poloxamer 407 (16 and 25%) to AX-HP NCs to prepare hydrogels with improved thermal stability and demonstrated a sustained in vitro release (50%) for a period of 72 h and 9-fold higher DPPH-based antioxidant activity than ascorbic acid, suggesting that the poloxamer 407-based AX-HP nanohydrogel could provide prolonged skin protection in alleviating photoaging and skin cancer through inhibition of the photooxidation process [104].

Likewise, several biopolymers such as chitosan oligosaccharides/PLGA, zein/casein and zein have been used respectively for nanoencapsulation of astaxanthin, fucoxanthin and lutein [95]. Compared to non-polymeric nanocarotenoids, the carotenoid NPs/NCs/NFs involving polymer/biopolymer were shown to be the most effective in enhancing storage stability and bioavailability. Nevertheless, there is a need to use natural biopolymers and innovative preparation techniques for exploring functional attributes and application of nanocarotenoids in the food/drug industry.

### 4.4. Solid Lipid Nanoparticles (SLNPs) and Nanostructured Lipid Carriers (NLCs) 

SLNPs are the lipid nanocarriers consisting of fatty acids or mono-, di-, or triglycerides, waxes and partial glycerides in solid form at room temperature, while NLCs contain a mixture of solid and liquid lipids/oils at room temperature as schematically shown in Figure 3E,F, respectively [3,9]. Depending on both composition and preparation conditions, the particle size of SLNPs and NLCs was reported to range from 10–1000 nm [105,106]. Both SLNPs and NLCs can be prepared by adopting different approaches such as high-energy and low-energy methods as well as organic solvent and lyophilization based methods [107]. High energy methods are traditionally used to produce food-grade SLNPs/NLCs by employing mechanical techniques involving high-pressure homogenization, cold and hot homogenization and high shear homogenization. Whereas, the low energy methods involve utilization of internal properties of the system including membrane contactor, phase inversion temperature, coacervation, double emulsion and microemulsion cooling technique. Additionally, the organic solvent based methods such as emulsification-solvent evaporation and diffusion techniques, solvent injection, supercritical fluid extraction, particle from gas saturated solution and gas-assisted melting atomization technique can be used as well [107]. Of the various methods, low energy methods are commonly used for preparation of SLNPs and NLCs as no solvent and expensive equipment are needed. Several advantages of using SLNPs and NLCs as nanocarriers include thermal stability, high loading capacity, ease of preparation, reduced drug mobility, enhanced bioavailability, molecular level interaction at the target site and low production cost [105].

Accordingly, SLNPs and NLCs have emerged as promising nanoscale systems for efficient nanoencapsulation and targeted delivery of carotenoids. For example, Tamjidi, et al. [108] prepared astaxanthin-loaded NLCs containing 555 mg of Tween 80, 20 mg of astaxanthin in oil, 757 mg of glyceryl behenate, 218 mg of oleic acid and 5 mg of lecithin, 100 ppm of ethylenediaminetetraacetic acid (EDTA) and 0.02% of sodium azide (*w/v*) by melt-emulsification and ultrasonication. The astaxanthin NLCs with a mean particle size at 94 nm and ZP at −24 mV exhibited high physical stability (30 days at 20 °C) in all the three beverage model systems including 12% sucrose at pH 3 and 7, semi-actual whey and actual non-alcoholic beer. The presence of sucrose could enhance the physical stability of astaxanthin NLCs in an acidic beverage model (pH 3), whereas both particle size and astaxanthin level remained unchanged in semi-actual whey. On the contrary, both carbonation and/or thermal pasteurization of astaxanthin NLCs-containing beer resulted in a rise in particle size and loss in astaxanthin. Nevertheless, the stability of astaxanthin NLCs in non-pasteurized CO_2_-free beer was improved at low storage temperature, implying that carotenoid NLCs may be applied to the beverage industry.

For β-carotene encapsulation, NLC-based nanocarriers were developed by Zirak and Pezeshki [109] by using a solid lipid/oil phase ratio of 10:1. More elaborately, β-carotene-NLCs with a particle size at 79–115 nm and ZP at 0.3–0.8 mV were prepared by dissolving β-carotene (0.03%) in octyl octanoate (0.3%), mixing with 3% compritol in a hot water bath at 85 °C, and incorporating 3% poloxamer 407 into lipid phase for homogenization at 20,000 rpm for 30 min. The β-carotene-NLCs was shown to be stable over a 60-day storage period at 25 °C. Later, two different studies have demonstrated that the preparation of β-carotene-SLNPs could enhance physicochemical stability of β-carotene. In the first study, Mehrad, et al. [57] developed β-carotene-SLNPs with a particle size <200 nm and ZP at 20–30 mV by dispersing 50 mg of β-carotene in a lipid phase containing 2 g of corn oil and 3 g of palmitic acid and stirring at 85 °C for 5 min, followed by adding into an aqueous phase containing 1% whey protein isolate in water (pH 6.9), and subjecting to high-shear homogenization for 3 min at 17500 rpm. Although the β-carotene-SLNPs prepared by this method was quite stable at low pH and low ionic strength, the β-carotene degradation increased following a rise in temperature. On the other hand, Schjoerring-Thyssen, et al. [58] prepared β-carotene-SLNPs by employing a hot-melt high-pressure homogenization method with the structure possessing a core triglyceride platelet surrounded by an amorphous β-carotene layer. More specifically, the aqueous phase containing 40 g of Tween 80, 10 g of sunflower lecithin and 350 g of deionized water was homogenized for 2 h at 25 °C, followed by mixing with sunflower oil, homogenizing at 18,000 rpm for 5 min at (70 °C) and subjecting to high-pressure homogenization at 800 bar for 6 cycles. The β-carotene-SLNPs containing 37.5% β-carotene was obtained with a particle size at 120 nm and ZP at −30 mV, and shown to be stable over a 20-month storage period at room temperature.

**Figure 11:**
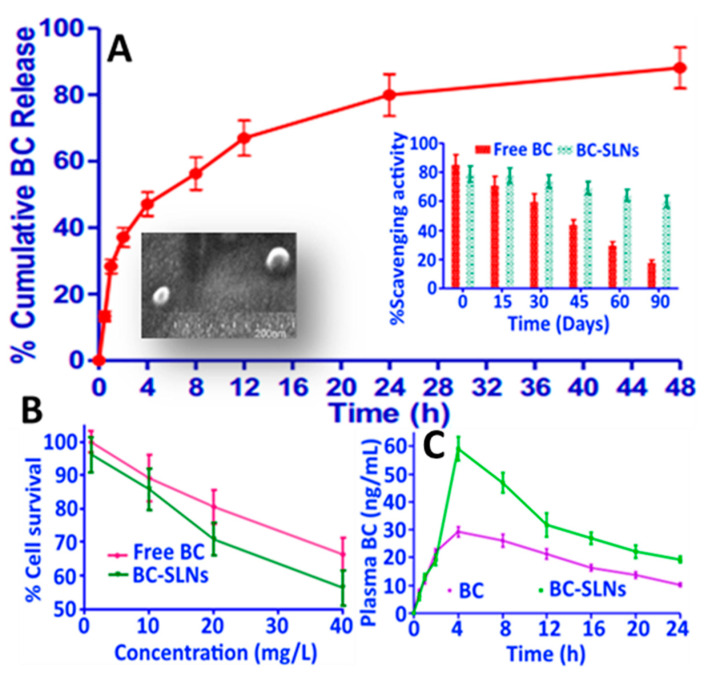
In vitro release profile of β-carotene from β-carotene solid lipid nanoparticles in 0.1 N hydrochloric acid along with two insets showing the scanning electron microscopic image of β-carotene solid lipid nanoparticles and antioxidant activity of free β-carotene and β-carotene solid lipid nanoparticles (**A**) as well as their anti-breast cancer activity towards MCF-7 cells after 48 h of incubation (**B**) and plasma β-carotene levels as a function of time after administering in mice (**C**). The results were expressed as mean ± standard deviation of ≥ 3 independent replicates and error bars represent the standard deviation from mean values. BC = β-carotene and SLNs = solid lipid nanoparticles. Adapted with permission from Jain, et al. [56].

In a later study, Jain, et al. [56] developed β-carotene-SLNPs by hot homogenization involving sequential mixing of 100 mg of β-carotene, 700 mg of glyceryl mono stearate/gelucire and 500 mg of ethanolic phospholipid, followed by adding the mixture to a heated aqueous solution containing Tween 80 (0.5%) and Pluronic F68 (0.1%), subjecting to homogenization for 30 min at 10,000 rpm and removing solvent by a dialysis bag for subsequent lyophilization. The β-carotene-SLNPs obtained with a particle size ranging from 200 to 400 nm, ZP from −6.1 to −9.3 mV and EE from 53.4 to 68.3% could enhance β-carotene stability over a 3-month storage period at 2–8, 25 and 40 °C. Also, the β-carotene-SLNPs showed a cumulative β-carotene release of 82% in 0.1 N hydrochloric acid (Figure 11A). Moreover, the antioxidant activity of β-carotene-SLNPs was higher than free β-carotene, as evidenced by a DPPH scavenging activity change from 80 to 60% for the former and from 82 to 18% for the latter over a 3-month storage period (Figure 11A). For anti-cancer effect, the β-carotene-SLNPs at 40 μM was more effective in inhibiting the growth of breast cancer cells MCF-7 (42%) than that of free β-carotene (32%) after 48 h incubation (Figure 11B), demonstrating that small-sized particles (~200 nm) can more efficiently evade phagocytosis to penetrate into cancer cells at a faster rate than the large-sized ones. Time profiles on mouse plasma β-carotene levels as affected by free β-carotene and β-carotene SLNs are shown in Figure 11C and the pharmacokinetic parameters AUC, C_max_, T_max_, t_1/2_, mean residence time for free β-carotene and β-carotene SLNs were 633.8 and 1215.3 µg/mL.h, 29.3 and 59.1 µg/mL, 4 and 4 h, 11.9 and 16.3 h, and 19.0 and 24.5 h, respectively (Figure 11C).

**Figure 12:**
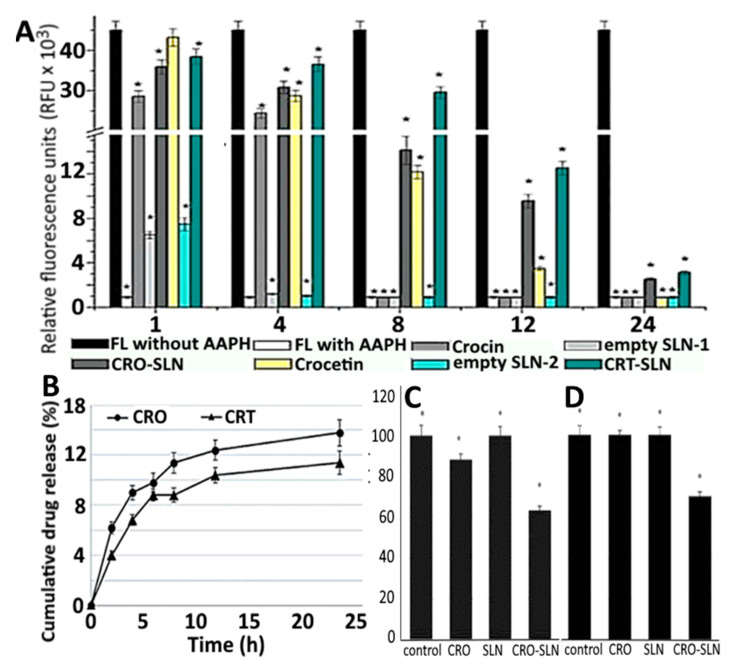
Oxygen radical absorbance capacity for free crocin and free crocetin as well as blank, crocin and crocetin solid lipid nanoparticles as measured by relative fluorescence units (**A**) along with cumulative crocin and crocetin release in phosphate buffered solution (**B**) and cell viability towards the human melanoma A375 cells (**C**) and malignant Schwann sNF96.2 cells (**D**). The results were expressed as mean ± standard deviation of ≥ 3 independent replicates and error bars represent the standard deviation from mean values. *, ** and *** above each bar indicating significantly different values. RFU = relative fluorescence unit, FL = fluorescein, AAPH = 2,2′-azobis(2-amidinopropane) dihydrochloride, CRO = crocin and CRT = crocetin. Adapted with permission from Puglia, et al. [110].

To increase the stability and antioxidant activity of carotenoids crocin and crocetin, Puglia, et al. [110] prepared SLNPs-based nanocarriers using quasi-emulsion solvent and solvent diffusion methods, respectively. Both crocin- and crocetin-SLNPs showed high stability and homogeneity with a mean particle size at 273.3 and 278.1 nm, polydispersity index at 0.16 and 0.31, ZP at 30.2 and 17.8 mV and EE at 82.1 and 94.0%, respectively. Based on the oxygen radical absorbance capacity assay, a higher antioxidant activity in terms of relative fluorescence units (RFU) was obtained for crocin-SLNPs (1000–35,000 RFU) and crocetin-SLNPs (2500–38,000 RFU) compared to unloaded SLNPs (Figure 12A). Also, an in vitro release of 68 and 60% using gastric fluids was shown for crocin-SLNPs and crocetin-SLNPs, respectively (Figure 12B), while both could inhibit growth of human melanoma cancer cell A375 (Figure 12C) and malignant Schwann cell sNF96.2 (Figure 12D).

Highly nonpolar carotenoid lycopene-based SLNPs were prepared by Nazemiyeh, et al. [59], who used a hot homogenization method by mixing the lipid phase containing 3.5% of lycopene, 43.9% of glyceryl palmitostearate (Precirol^®^ ATO5), 6.1% of Tween 80 and myristic acid (< 0.5%) with the hot aqueous phase containing 46.6% of Poloxamer 407, followed by homogenizing at 12000 rpm for 30 min at 70 °C. The lycopene-SLNPs showed a mean particle size of 125 nm, polydispersity index of 0.16, ZP of −10.1 mV, EE of 98.4% and drug loading of 44.8%. No significant change in these physicochemical characteristics was observed after 3-month storage at 4 °C, indicating that an improved stability of lycopene SLNPs was attained. A similar particle size stability over a 120-day storage period at 4, 30 and 40 °C was also shown by Okonogi and Riangjanapatee [8], preparing lycopene NLCs by hot high-pressure homogenization (75 °C for 3 cycles) of a mixture of lipid phase (100 g of lycopene-loaded NLC suspensions in 5 g melted lipid of orange wax and rice oil at 9:1 ratio) and aqueous phase containing 1 g of Eumulgin^®^SG in 90 mL water. However, the ZP value declined with storage time at 4 and 30 °C. The particle size, ZP and EE of as-prepared lycopene-NLCs ranged from 157 to 166 nm, −74.2 to −74.6 mV and EE (>99%). In a later study Singh, et al. [60] also demonstrated a similar stability over a 120-day period at 4, 30 and 40 °C for lycopene NLCs without any noticeable precipitation or phase separation. Moreover, a prolonged shelf-life and good dispersibility was shown over a 324-day storage period at room temperature. Such highly stable lycopene NLCs with a spherical shape, particle size at 121.9 nm, ZP at −29 mV, EE at 84.5% and drug loading at 9.5% were obtained by ultrasonication of a mixture containing 12% of Precirol^®^ ATO5, 5% of surfactant mixture (Tween 80 and Poloxamer 188 at 1:2) and 45 s of sonication time. The lycopene NLCs showed a sustained in vitro release over 48 h, high antioxidant activity in terms of DPPH (184.2 μg Trolox) and ABTS (143.1 μg Trolox) scavenging activity, as well as enhanced *ex vivo* gut permeation and improved cytotoxicity towards human breast cancer cell MDA-MB231, compared to lycopene extract.

### 4.5. Supercritical Fluid-Based Nanoparticles

Supercritical fluids (SCFs) are the substances that exhibit both liquid- and gas-like properties at a critical temperature and pressure with carbon dioxide being the most commonly used in food and drug applications owing to its several ideal properties including non-inflammable, non-toxic, inert, low critical point (*T*_c_ = 31.1 °C and *P*_c_ = 73.8 bar), inexpensive and easily removable from food matrix [2]. Also, it can produce high purity NPs with homogeneous drug distribution. In general, the preparation of NPs by SCF-CO_2_ method involves mixing polymer and drug in SCF-CO_2_, followed by precipitation and extrusion through a tiny nozzle [111]. The production of carotenoid NPs by SCF-CO_2_ can be routinely performed by five different methods, namely, rapid expansion of supercritical fluids (RESF), gas anti-solvent (GAS), supercritical anti-solvent (SAS), particles from gas-saturated solutions (PGSS) and supercritical extraction from an emulsion (SFEE) [2]. Some other supercritical fluid-based techniques including solution enhanced dispersion by supercritical fluids (SEDS), aerosol solvent extraction system (ASES), polymerization-induced phase separation (PIPS), supercritical solvent impregnation (SSI) and supercritical assisted atomization (SAA) can also be used. However, in recent years, studies on nanoencapsulation of carotenoids by SCF method have mainly focused on SEDS-based SCF method and some of them are reviewed as shown below.

**Figure 13:**
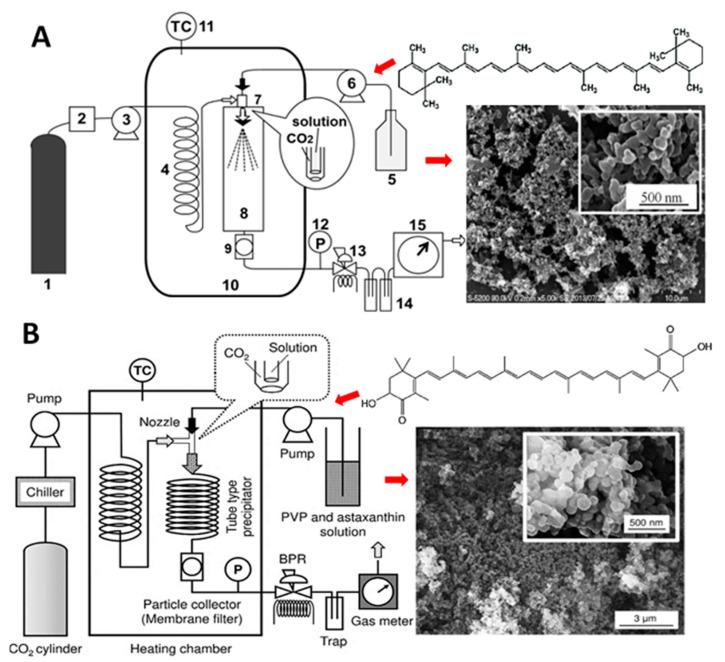
Schematic diagram of the solution enhanced dispersion by supercritical fluid method used for preparation of β-carotene solid lipid nanoparticles (**A**) and astaxanthin solid lipid nanoparticles (**B**) along with each panel showing the respective carotenoid structure and scanning electron micrographic image of solid lipid nanoparticles. In (**A**), steps 1 to 15 represent different processing steps involved in solution enhanced dispersion by supercritical fluid method. Adapted with permission from Kaga, et al. [61] and Nerome, et al. [112].

Nerome, et al. [112] prepared β-carotene NPs by SEDS method through pumping β-carotene (1.5 mg/mL) in different solvents (dichloromethane, *n*-hexane, ethyl acetate or *N*,N-dimethylformamide) at a flow rate of 0.25 mL/min into a precipitator under different pressure (8–12 MPa) and temperature (40–60 °C) conditions. SCF-CO_2_ was simultaneously pumped at 20 mL/min for mixing with β-carotene solution and extrusion through a coaxial nozzle (Figure 13A). Upon diffusion of SCF-CO_2_ into organic solvent, a supersaturation of β-carotene occurred, resulting in precipitation of irregularly-shaped NPs (135 nm) by using ethyl acetate as solvent at an optimized pressure of 12 MPa and temperature of 40 °C. In a later study, Kodama, et al. [75] produced high content of Z-lycopene isomers (97.8%) by thermal isomerization (80 °C, 8 h) and filtration of purified all-*E*-lycopene (Figure 3G) for preparation of NPs by SEDS method and reported that NPs with a much smaller size could be obtained with high level of Z-lycopene isomers (75 nm) than that with all-*E*-lycopene isomers (3.6 μm). More elaborately, lycopene dissolved in ethyl acetate was pumped at a flow rate of 0.5 mL/min for 3 h and mixed with SCF-CO_2_ (flow rate, 15 mL/min) at 40 °C and 10 MPa for extrusion through a 0.4-mm coaxial nozzle for formation of NPs. 

Similarly, Kaga, et al. [61] produced *Z*-isomer-rich astaxanthin by thermal isomerization and filtration of all-*E*-astaxanthin and prepared water-soluble *Z*-astaxanthin NPs with polyvinylpyrrolidone (PVP/Z-AX NPs) by using the SEDS method (Figure 13B). Through simultaneous pumping of 19:1 (*v/v*) acetone/ethanolic solution of PVP/Z-AX (0.1 mL/min for 1 h, 10 MPa, 60 °C) and SCF-CO_2_ (15 mL/min), the PVP/Z-AX NPs with a particle size from 150–175 nm were obtained. However, by raising the PVP ratio, the particle size decreased with an optimum PVP:astaxanthin ratio being at 10:1. Moreover, the astaxanthin content increased following a rise in temperature or decline in pressure. The authors postulated that *Z*-isomer of astaxanthin, being more effective to coprecipitate than its all-*E*-isomer counterpart during preparation using the SEDS method, could exhibit the highest bioavailability and antioxidant capacity. However, in all the above three studies, the stability of carotenoid NPs remained unexplored. Nevertheless, the SEDS method can be used for preparation of NPs from *Z*-isomer-enriched carotenoids obtained through thermal pretreatment and filtration. 

More recently, the ethyl cellulose-based astaxanthin NPs were prepared from astaxanthin standard and an o/w emulsion obtained by mixing an oil phase containing ethyl cellulose and ethyl acetate with an aqueous phase containing ethyl acetate-saturated water and Tween 80, followed by sonicating and passing through a continuous supercritical emulsion extractor with pressure at 8 MPa, temperature at 311 K, liquid/gas ratio at 0.1 and SCF-CO_2_ flow rate at 1.4 kg/h [62]. The ethyl cellulose-based astaxanthin NPs in both spherical and elliptical shape was obtained with a mean particle size of 266 nm, EE of 84% and astaxanthin loading of 21 mg/g, while it exhibited 70% in vitro release in simulated intestinal fluid and high antioxidant activity with Trolox equivalent at 3900 M, suggesting that the continuous supercritical emulsion extraction method can be an ideal technique for preparation of astaxanthin NPs. Compared to conventional methods, the utilization of SCF techniques for nanoencapsulation of carotenoids has several advantages, including low-temperature operation, single-step processing and ability to produce solvent-free and homogenous products. Thus, the SCF techniques should be more appropriate for production of high-value added products. However, the application of a wide variety of SCF methods in nanoencapsulation of functional foods, drugs, micronutrients and nutraceuticals still needs to be explored.

### 4.6. Metal/metal Oxide-Based Nanoparticles and Hybrid Nanocomposites 

**Figure 14:**
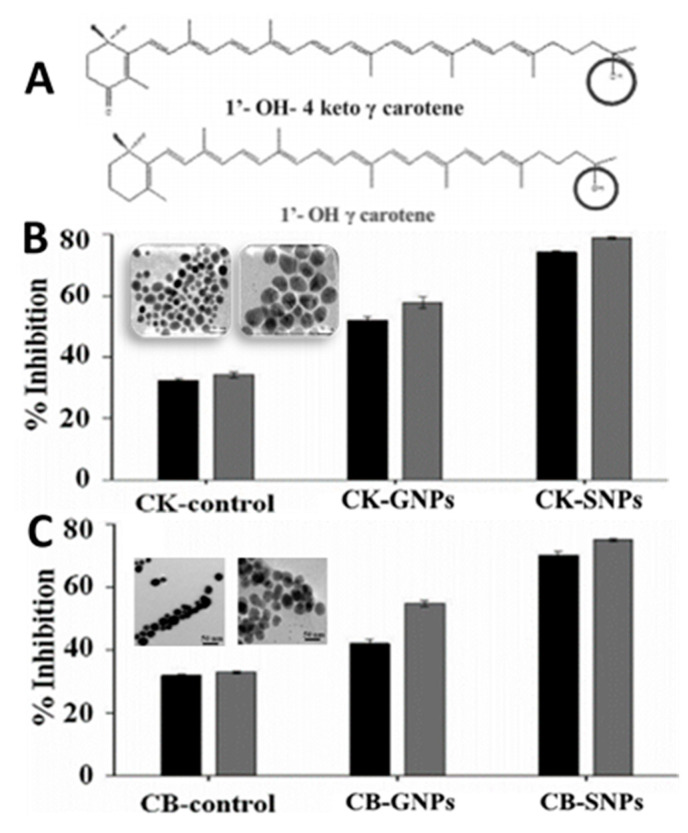
Chemical structures of 1′-OH-4 keto γ-carotene and 1′-OH γ-carotene (**A**) used for synthesis of gold nanoparticles and silver nanoparticles along with their nitric oxide and 2,2-diphenylpicrylhydrazyl free radical scavenging activity shown by 1′-OH-4 keto γ-carotene based gold and silver nanoparticles (**B**) as well as 1′-OH γ-carotene based gold and silver nanoparticles (**C**) compared to the control. The results were expressed as mean ± standard deviation of ≥3 independent replicates and error bars represent the standard deviation from mean values. GNPs = gold nanoparticles, SNPs = silver nanoparticles, CK = 1′-OH-4 keto γ-carotene, CB = 1′-OH γ-carotene. 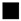 and 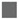 represent bars corresponding to nitric oxide and 2,2-diphenylpicrylhydrazyl scavenging percentage values, respectively. Adapted with permission from Sowani, et al. [115].

Metal/metal oxides nanoparticles are among the most widely used nanomaterials possessing unique properties and can be synthesized by physical, chemical and biological means, with the first one employing a top-down approach, while the latter two adopting a bottom-up process [113]. In recent years, by taking into account the safety and availability of natural raw materials and versatility in metabolite type, the synthesis of metal/metal oxides nanoparticles by green methods has drawn considerable attraction [114]. For example, Sowani, et al. [115] synthesized both gold and silver NPs (GNPs and SNPs) by incubating with *Gordonia amicalis* HS-11 containing two carotenoid pigments 1′-OH-4-keto-γ-carotene and 1′-OH-γ-carotene at pH 9 and 25 °C for 24 h, which were shown to possess metal-reducing properties and the synthesized GNPs and SNPs could effectively scavenge both NO (69.7%) and DPPH (47.9%) in vitro, respectively (Figure 14A–C). Similarly, by using the aqueous extract of Sumac, Shabestarian, et al. [65] synthesized spherically-shaped GNPs under continuous stirring at 40 °C for 40 min for evaluation of antioxidant activity. Sumac-GNPs with a mean particle size of 20.83 nm and ZP of −25.30 mV were demonstrated to exhibit high DPPH (96.8%) and ABTS (85.7%) free radical scavenging activity at a concentration of 800 µM. In another study Patra and Baek [63] synthesized GNPs with a particle size ranging from 20 to 140 nm by mixing the aqueous extract of water melon rind (*Citrullus lanatus*) with 1 mM auric chloride. The as-synthesized *C. lanatus*-based GNPs showed good antibacterial activity against *Bacillus cereus*, *Listeria monocytogenes*, *Staphylococcus aureus*, *Escherichia coli* and *Salmonella typhimurium* with zone of inhibition ranging from 10.3 to 24.8 mm as well as proteasome inhibition (28.2%) and high antioxidant properties in terms of scavenging DPPH (24.7%), NO (25.6%) and ABTS (29.4%).

**Figure 15:**
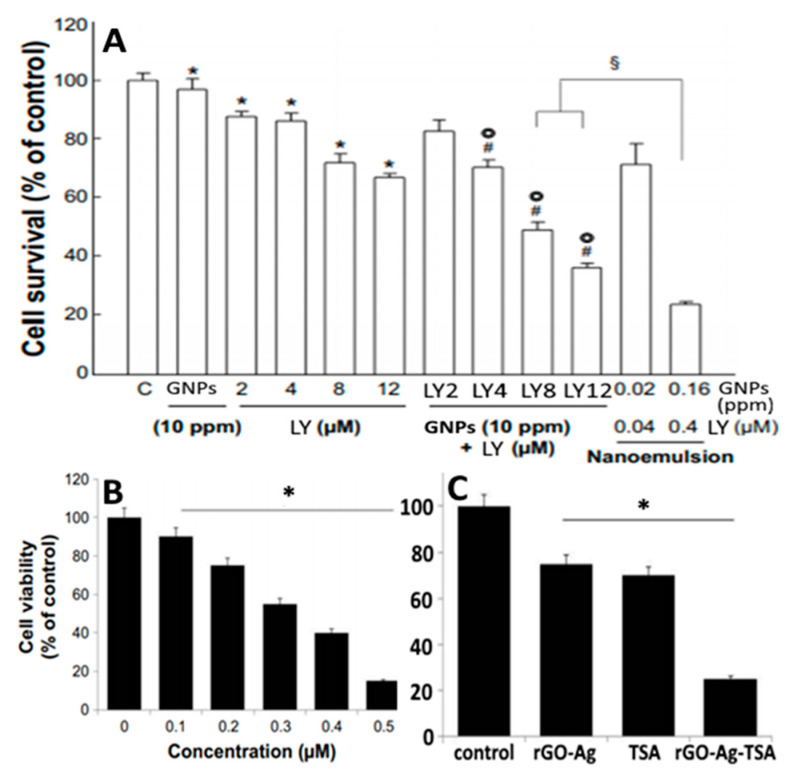
In vitro anti-colon cancer activity and anti-ovarian cancer activity exerted, respectively, by lycopene-gold nanoparticles based nanoemulsion towards HT-29 cells (**A**) and lycopene reduced graphene oxide silver nanoparticles towards SKOV3 cells (**B**). In (**A**), inhibitory effects of different doses of commercial gold nanoparticles and lycopene alone or in combination as well as lycopene-gold nanoparticle nanoemulsion on HT-29 cells after incubation for 24 h. In (**B**), cell viability of SKOV3 cells upon treatment with different doses of lycopene reduced graphene oxide silver nanoparticles (0–0.5 μM) (**B**), while (**C**) showing comparison of cell viability upon treatment with rGO-Ag alone (0.2 μM), trichostatin A alone (0.2 μM) and both rGO-Ag and trichostatin A in combination (0.2 μM each). The results were expressed as mean ± standard deviation of ≥3 independent replicates and error bars represent the standard deviation from mean values. *, ^⌾^, #, or § symbols above each bar denote significantly different values. LY = lycopene, GNPs = gold nanoparticles, rGO-Ag = reduced graphene oxide-silver nanoparticle and TSA = trichostatin A. Adapted with permission from Huang, et al. [64] and Zhang, et al. [116].

Several carotenoids such as lycopene, crocin and canthaxanthin were used as both reducing and/or stabilizing agents for synthesis of carotenoid-functionalized GNPs and SNPs. Lycopene NEs carrying GNPs (LN-GNPs) were prepared by Huang, et al. [64] for evaluation of their anti-colon cancer activity against HT-29 cells. The LN-GNPs composed of lycopene (41.6 µg/mL) and GNPs (51 µg/mL) were prepared by mixing the oil phase containing lycopene (1.7 mg) and Tween 80 (1.2 g) with the aqueous phase containing deionized water (3.2 mL) and 3–5 nm GNPs (7.6 mL), followed by sonicating for 1 h. Spherically-shaped LN-GNPs with a mean particle size of 25 nm and ZP of −32.2 mV were demonstrated to possess high stability upon storage at 4 and 25 °C for 3 months as well as heating at 100 °C for 4 h. In addition, the LN-GNPs were more effective in inhibiting HT-29 cells than lycopene extracts probably caused by passive targeting effect (Figure 15A). In a later study, the lycopene-reduced graphene oxide-silver NPs (LGOSNPs) with spherical shape and particle size ranging from 10–50 nm were prepared by mixing 100 mg of graphene oxide with 60 mL water, followed by sonication for 60 min, successive addition of 30 mL of 1 mM AgNO_3_ and 10 mL of 5 μM lycopene, and stirring at 90 °C for 12 h. The as-synthesized LGOSNPs could exert anti-ovarian cancer activity with an IC_50_ value of 0.3 μM (Figure 15B) and by mixing LGOSNPs and trichostatin A at 0.2 μM each, a synergistic effect was shown to occur (Figure 15C) [116]. In another study, Hoshyar, et al. [117] prepared spherical-shaped GNPs with a particle size ranging from 4–10 nm by one-pot green synthesis using crocin as both reducing and stability agent. The crocin-GNPs, synthesized by an optimized condition with 1 mL of 1 mM HAuCl_4_, 6 mL of 682.4 mM crocin, reaction time of 24 h and temperature of 50 °C, were shown to be stable over a 2-month storage period at 25 °C and exhibit anti-breast cancer activity with 50% reduction in cell viability. More recently, Venil, et al. [118] used the ethanolic extract of bacterium *Dietzia maris* AURCCBT01 for synthesis of canthaxanthin-mediated SNPs. By mixing 10 mL of 1 mM silver nitrate with 1 mL of canthaxanthin extract, followed by incubating for 30 min at room temperature, the SNPs with a particle size at 40–50 nm were obtained and shown to effectively inhibit the growth of human keratinocyte HaCaT cell line with an IC_50_ at 43 µg/mL. Besides GNPs and SNPs, zinc oxide nanotubes were also used for encapsulation of carotenoids. Based on a theory of molecular dynamics and density functional simulations, Monteiro, et al. [76] studied the stabilization dynamics of β-carotene encapsulated zinc oxide nanotubes (14.1 × 49.1 Å) and illustrated that the encapsulation is an energetically-favorable process with β-carotene being close to zinc oxide nanotubes wall and their interaction leading to charge delocalization from β-carotene to zinc oxide nanotubes (Figure 3H).

## 5. In Vitro Release, Gastrointestinal Absorption and Bioaccessibility/Bioavailability

**Figure 16:**
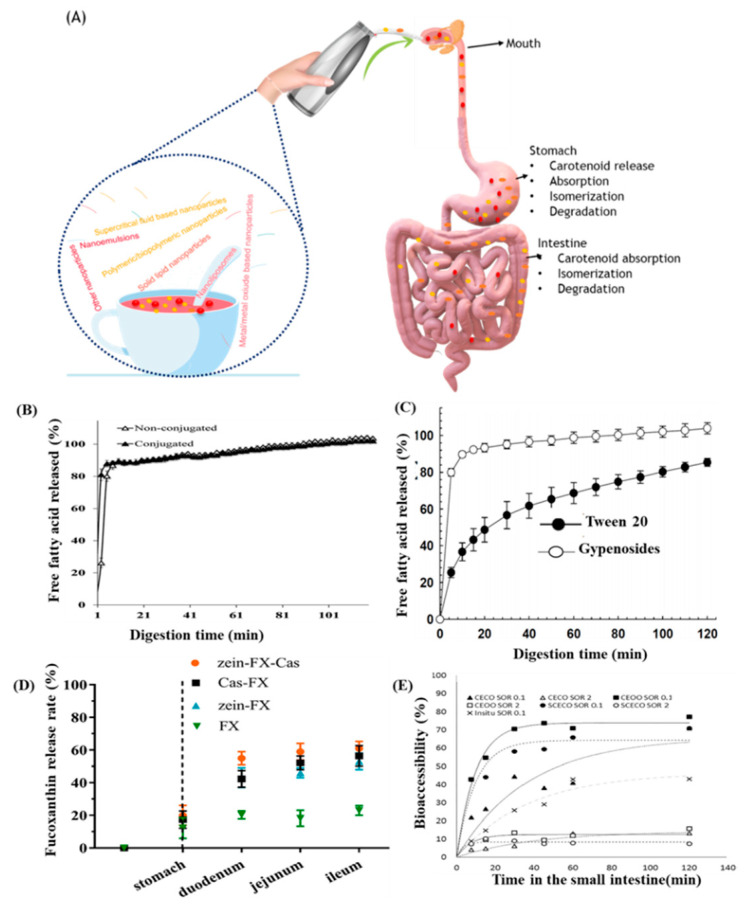
Selected in vitro digestion studies of nanoencapsulated carotenoids. A schematic on gastrointestinal absorption of nanoencapsulated carotenoids (**A**), free fatty acid release behavior of lutein-loaded nanoemulsions stabilized with non-conjugated sodium caseinate and Maillard conjugates (**B**), free fatty acid release behavior of astaxanthin-loaded nanoemulsions using gypenosides and Tween 20 (**C**), nanoencapsulated fucoxanthin (FX) release rate using different stabilizers (**D**) and bioaccessibility (%) of six oils enriched with β-carotene (**E**). The results were expressed as mean ± standard deviation (SD) of ≥3 independent replicates. Cas = caseinate, CE-CO = carrot-enriched corn oil, CE-OO = carrot-enriched olive oil, SCE-CO = β-carotene-enriched corn oil and SOR = surfactant-to-oil ratio. Adapted with permission from Chen, et al. [83], Gumus, et al. [120], Li, et al. [121], and Salvia-Trujillo, et al. [122].

Bioefficiency of a nanoencapsulated material is a vital criterion to meet the metabolic needs of a consumer. Quantitative estimation of bioefficiency is usually done by determination of bioaccessibility, bioavailability and biological activity of consumed food components [119]. Bioavailability of a compound is defined as the fraction of functional component absorbed and reaching the systemic blood circulation for performing physiological functions and/or biodistribution (Figure 16A). Accordingly, the oral bioavailability of an administered compound can be expressed according to following Equation (1).

(1) $$\mathrm{Bioavailability}=F_{C}\times F_{B^{*}}\times F_{A^{*}}\times F_{T^{*}}$$

where F_C_ is the fraction of a bioactive component from food to ingestion, F_B*_ is the fraction of an ingested bioactive component accessible to intestinal absorption (bioaccessibility), F_A*_ is the fraction of a bioactive component absorbed through the intestinal wall and F_T*_ is the fraction of a bioactive component remaining after chemical and enzymatic transformations for entering systemic circulation and biodistribution. 

Unlike bioavailability, bioaccessibility is the fraction of an ingested bioactive component that becomes accessible for absorption through the epithelial layer of gastrointestinal tract and can be determined using the following Equation (2).

(2) $$\mathrm{Bioaccessibility}\;\left(\%\right)=\left[\frac{\mathrm{The}\;\mathrm{amount}\;\mathrm{of}\;a\;\mathrm{biocomponent}\;\mathrm{solubilized}}{\mathrm{The}\;\mathrm{amount}\;\mathrm{of}\;a\;\mathrm{biocomponent}\;\mathrm{in}\;\mathrm{raw}\;\mathrm{digested}}\right]$$

Bioaccessibility is usually determined in vitro by simulating gastric condition with pepsin/HCl (pH 1–2) and intestinal condition with pancreatin/bile salt or trypsin (pH 7–7.5), followed by mimicking an absorption model using Caco-2 cell or dialysis bag [42,47,56]. Whereas, the absolute bioavailability in vivo is determined by simultaneous oral administration by gavage and intravenous injection into rats, followed by blood collection and bioactive component analysis at different time intervals and determination of pharmacokinetic parameters including AUC (area under the curve of bioactive component concentration *versus* time profile), C_max_ (maximum concentration in blood), T_max_ (time to reach maximum concentration in blood) and t_1/2_ (time to reach half the maximum concentration) [44]. Consequently, the bioactive component carrier needs to be highly water-soluble to attain high bioaccessibility and bioavailability for enhancement of biological activity.

Gumus, et al. [120] developed casein coated and casein-dextran coated lutein NEs for evaluating their gastrointestinal stability and bioaccessibility in the presence of Maillard conjugates, obtained by spray drying at 60 °C for 48 h. Following a rise in temperature, both lutein NE-types showed a slight increase in aggregation at temperature >37 °C, accompanied by color fading due to lutein degradation. Compared to casein coated lutein NEs, the casein-dextran coated one was more stable at pH 3–7 and under simulated gastric condition, probably due to steric repulsion by dextran moiety. Also, there was no significant difference in free fatty acid release percentage after digestion (Figure 16B) and bioaccessibility (8.2%), implying that Maillard conjugates may enhance the physical stability of lutein NEs without affecting the bioaccessibility.

To improve the in vitro small intestinal digestion and bioaccessibility of astaxanthin NEs, Chen, et al. [83] used gypenosides as a stabilizer to prepare astaxanthin NEs by mixing the aqueous phase containing astaxanthin (95%, *w/w*) with the oil phase (5%, *w/w*) and subjecting to high-pressure homogenization at 100 MPa for 4 cycles. Compared to Tween 20-stablized astaxanthin NEs, the gypenosides-stabilized astaxanthin NEs with a particle size at 125 nm and ZP from −13.6 to −37.8 mV was shown to be stable over the pH range 6–8 and thermal treatment from 60–120 °C. Likewise, the gypenosides-stabilized astaxanthin NEs could more effectively protect astaxanthin from degradation during storage at 5 or 25 °C. However, gypenosides could result in a lower lipid digestion and astaxanthin bioaccessiblity than Tween 20 as evidenced by a lower free fatty acid release after digestion (Figure 16C). 

By employing a simple antisolvent precipitation method, Li, et al. [121] developed caseinate-stabilized zein NPs (CSZ-NPs) to encapsulate fucoxanthin through hydrophobic interaction at neutral pH. The fucoxanthin-loaded CSZ-NPs obtained with a particle size at 150 nm and ZP from −30 to −35 mV were stable during heating at 75 °C for 150 min and storage for 30 days at 25 °C. Compared to free fucoxanthin, fucoxanthin encapsulated in CSZ-NPs showed higher ABTS radical scavenging activity and a burst fucoxanthin in vitro release of 53.1–61.0% after 6 h digestion. Also, the fucoxanthin loaded in CSZ-NPs showed the highest in vitro release, followed by caseinate and zein stabilized NPs (Figure 16D).

Ravi and Baskaran [50] developed a fucoxanthin-loaded chitosan-glycolipid hybrid nanogels (FX-CH-GL NGs) for improved carotenoid stability and bioaccessibility. By using an ionic gelation method, FX-CH-GL NGs composed of chitosan and glycoside (1:0.5, *w/w*), sodium tripolyphosphate in water (0.03%) and fucoxanthin (1 mg) was prepared with a particle size at 200–500 nm, ZP at 30-50 mV and EE at 47–90%. The FX-CH-GL NGs were demonstrated to possess high fucoxanthin stability (t_1/2_, 45 h) and enhance bioavailability in vitro (68%) compared to that of the standard fucoxanthin (51%) and fucoxanthin + glycolipid (35.5%).

Salvia-Trujillo, et al. [122] prepared β-carotene NEs with a particle size at 226–279 nm and ZP at −23 mV by mixing β-carotene-enriched oil (carrot purée: 204.7 µg/g; synthetic β-carotene: 192.7 µg/g) with Tween 80 at a ratio of 2:1, followed by homogenization at 100 MPa for 3 cycles. An in vitro lipid digestion kinetics study revealed a complete inhibition of lipid hydrolysis with low bioaccessibility ranging from 8.4–14.4% for carotenoid loaded in different oils (Figure 16E), implying that the NEs prepared with a surfactant level (2%) can entrap carotenoids in undigested oil droplets, thereby preventing lipolysis and lowering bioaccessibility.

For determination of the in vitro bioaccessibility and bioavailability, Xia, et al. [123] prepared β-carotene loaded NEs by mixing 4% of β-carotene in olive oil or flaxseed oil with the 96% aqueous phase containing Tween 20 for stirring at room temperature for 1 h, followed by homogenization for 2 min and filtration through a high-pressure microfluidizer at 9000 psi for three cycles. By employing in vitro digestion models, the β-carotene loaded NEs obtained with a particle size at 178.1–183.5 nm and ZP at 39.9–41.9 mV showed a bioaccessibility of 47.8% and 65.2% for β-carotene loaded NEs prepared with flaxseed oil and olive oil, respectively. This may be explained as follows: compared to flaxseed oil, the olive oil-based β-carotene loaded NEs were digested more efficiently, generating more free fatty acids capable of forming mixed micelles, which in turn stimulated the formation of lipoprotein particles acting as a transcellular carrier of β-carotene through the intestinal epithelium, resulting in improved bioavailability. Furthermore, olive oil can form a larger chylomicrons and very low-density lipoproteins than flaxseed oil, suggesting that the difference in the fatty acid composition between olive oil and flaxseed oil may influence the formation of lipoprotein particles after gastrointestinal digestion. Thus, olive oil is more efficient in intestinal uptake of bioactive compounds and formation of chylomicrons in the intestinal epithelium.

In another study, Zhao, et al. [39] prepared and compared lycopene o/w NEs by using three vegetable oils (sesame oil, linseed oil or walnut oil) in terms of stability and bioaccessiblity. The lycopene NEs with a particle size at 200.1–287.1 nm and EE at 61.0–89.1% were obtained by mixing 0.2% of lycopene in oil with 2% of the emulsifier lactoferrin and subjecting to high pressure homogenization at 10,000 psi for 3 cycles. Sesame oil-based lycopene NEs showed the highest stability due to the high density as well as low viscosity and unsaturation of sesame oil as compared to the other two oils. Also, by employing a simulated gastrointestinal model, the bioaccessibility of lycopene was greatly enhanced in the NE system, amounting to 18% for the walnut oil-based lycopene NEs and 25% each for both sesame oil- and linseed oil-based lycopene NEs. Likewise, several studies have also demonstrated an improved in vitro release behavior and bioaccessibility of nanoencapsulated β-carotene, lycopene, astaxanthin and fucoxanthin compared to their nonencapsulated counterparts [124,125,126,127].

Likewise, by using a dialysis bag method, Jain, et al. [56] performed an in vitro release study of β-carotene-SLNPs prepared by mixing β-carotene (100 mg), glyceryl mono stearate (700 mg), gelucire (700 mg) and phospholipid (500 mg), and reported an initial burst release (28.3%) for 1 h, followed by a slow and sustained release over 48 h. The faster β-carotene release from the surface or first layer of β-carotene-SLNPs should account for the initial burst effect, while a slow diffusion through the lipid matrix may be responsible for the slow release thereafter. Moreover, gelucire, a component used for preparation of β-carotene-SLNPs was reported to possess faster dissolution property, thereby facilitating the β-carotene release. In addition, the bioavailability in vivo was determined by administering β-carotene and β-carotene-SLNPs orally at a dose of 15 mg/kg body weight into Albino Wistar rats and collecting blood at different time intervals (0.5–24 h) for β-carotene analysis. The pharmacokinetic data showed a pronounced increase with the AUC_total_ for β-carotene-SLNPs being 1.9-fold higher than that for β-carotene, and thus the bioavailability of β-carotene-SLNPs was improved substantially.

In a later study, Han, et al. [48] prepared electrospun lutein loaded NFs (ES-LU-NFs) by mixing PVA/sodium alginate and lutein, stirring for 5 h at room temperature and vacuum drying to remove solvents. The ES-LU-NFs were further crosslinked with glutaraldehyde/saturated boric acid and then vacuum dried at room temperature for 12 h to obtain the crosslinked ES-LU-NFs with a particle size at 240–340 nm, EE at 91.9% and contact angle at 33.5–78.2. Consequently, the ES-LU-NFs showed an enhanced stability and hydrophilicity for sustained release up to 48 h.

## 6. Nanocarotenoids Application in Pharmaceutical, Nutraceutical and Food-An Overview

Nanoencapsulation of carotenoids has been rapidly growing especially in the field of pharmaceutical, nutraceutical, and food applications. In pharmaceutical industry, carotenoid nanoencapsulation is becoming popular in the production and optimization of carotenoid-based drugs for controlled release. For instance, Jain, et al. [100] reported a controlled delivery of zein nanoparticles for enhancement of pharmacokinetics of β-carotene and application as a breast cancer drug. More recently, the carotenoid loaded nanocariers such as gold nanoparticles, nanoemulsions and nanoliposomes were demonstrated to improve the efficiency of nanoencapsulated carotenoids for treatment of cancer [128]. Interestingly, among 250 different commercialized nanoparticle delivery systems reported by Ghaffari and Dolatabadi [129], 15% of them were directed towards pharmaceutical market with some commercialized nanocarotenoid products including a water-soluble nanoencapsulated β-carotene NanoCarotene^®^ from Inventa Technologies Private Limited, Singapore. On the other hand, there is an increasing demand for application of nanoencapsulated carotenoids in nutraceutical and functional food production as well as bioactive food fortification. More recently, nanocarotenoid products are increasingly evaluated for their application in fruit and nutrition beverages to enhance stability, absorption and bioavailability of carotenoids. For example, Akhoond Zardini, et al. [130] fortified nanocapsulated lycopene in orange drink and observed no change in sensory attributes, stating that such nanoencapsulation of lycopene can enrich foods with bioactive properties. Thus, the nanoencapsulation of carotenoids can facilitate a range of benefits to both nutraceutical and food applications including targeted delivery and nutrient absorption with acceptable sensory properties. While most studies have focused mainly on in vitro application of nanocarotenoids, their translation from bench to bedside remains inadequate, implying the key challenges involved in clinical trials and premarketing evaluation need to be overcome.

## 7. Conclusions and Future Prospective

In this review article, recent advancements in the field of carotenoid nanoencapsulation into a wide variety of nanostructures including NEs, NLs, NCs, NFs, NPs, SLNPs, NLCs and supercritical fluid-based nanoparticles were comprehensively reviewed. Many investigations are still semi-empirical and thus rational scale-up without compromising functional characteristics and bioactivities is required for possible large-scale production and broader application. Also, most studies mainly focused on optimization of preparation methods and nanostructure characterization to improve storage and thermal stability. However, there is still a paucity of data in elucidating both in vitro and in vivo release mechanisms. Therefore, there is an urgent need for more comprehensive studies by developing novel preparation techniques for subsequent evaluation of gastrointestinal digestion, absorption, release mechanism, bioaccessibility and cellular antioxidant activity, as well as anti-cancer inhibition mechanisms towards different types of cancer cells. More in vivo studies are also necessary for determination of pharmacokinetics and bioavailability, as well as treatment of chronic disease and tumor inhibition. The determination of biological activity of nanocarotenoids should also address the toxicology issues for future clinical application. In lieu of this, the research at a comprehensive and collaborative level is important for development of safe and cost-effective nanocarotenoids for long-term food and drug applications in the industry.

## Figures and Tables

**Table 1:** Nanosystems for encapsulation of carotenoids ^1^.

Nanosystem	Carotenoids	Particle Size (nm)	EE (%)	Zeta Potential (mV)	Storage Stability (Days)	References
Nanoemulsions	β-carotene	218	NA	40	21 at 37 °C	[32]
		143.7		−38.2	30 at 25 °C	[33]
	Microbial carotenoids	142.1		NA	30 at 25 °C	[34]
	Carotenoids	290 to 350		−53.4 to −58.8	21 at 25 °C	[35]
	β-carotene	198.4 to 315.6		−29.9 to −38.5	90 at 4, 25, and 37 °C	[36]
	Carotenoids	<200		−30 to −45	35 at 25 °C	[37]
	Lycopene	145.1 to 161.9		−19.7 to −20.7	1 at 25 °C	[38]
		200.1 to 287.1	61 to 89.1	20 to 45	42 at 4, 25, and 37 °C	[39]
Polymeric/biopolymeric NPs	Carotenoids	153	83.7	NA	NA	[40]
		84.4	>96	−41.3 to −43.6	60 at 41 °C	[41]
	β-carotene	77.8 to 371.8	98.7 to 99.1	−37.8 to −29.9	NA	[42]
	β-carotene	70.4	97.4	NA	NA	[43]
	Lycopene	152	89	58.3	NA	[44]
		~ 200	>95	−36	210 at 5 °C	[45]
		193	NA	−11.5	14 at 25 °C	[46]
	Lutein	<250	74.5	−27.2	NA	[47]
	Lutein	240 to 340	~91.9	NA	NA	[48]
	Crocetin	288 to 584	59.6 to 97.2	NA	NA	[49]
	Fucoxanthin	200 to 500	47 to 90	30 to 50	6 at 37 °C	[50]
Nanoliposomes/liposomes	Carotenoids	70 to100	75	−5.3	NA	[51]
	β-carotene	162.8 to 365.8	~98	64.5 to 42.6	70 at 4 °C	[52]
	Astaxanthin	80.6	97.6	31.8	15 at 4 and 25 °C	[53]
		60 to 80	97.4	NA	NA	[54]
	Lutein	264.8 to 367.1	91.8 to 92.9	−34.3 to −27.9	NA	[55]
SLNPs and NLCs	β-carotene SLNPs	200 to 400	53.4 to 68.3	−6.1 to −9.3	90 at 5, 25, and 40 °C	[56]
		<220	NA	20 to 30	10 at 25 °C	[57]
		120	NA	−30	56 at 25 °C	[58]
	Lycopene SLNPs	125 to 166	86.6 to 98.4	NA	60 at 4 °C	[59]
	Lycopene NLCs	157 to 166	> 99	−74.2 to −74.6	120 at 4, 30, and 40 °C	[8]
		121.9	84.50	−29	90 at 25 °C	[60]
Supercritical fluid-based NPs	Astaxanthin	150 to 175	NA	NA	NA	[61]
		266	84	NA	NA	[62]
Metal/metal oxide-based NPs and hybrid nanocomposites	Carotenoids	20 to 140	NA	NA	NA	[63]
	Lycopene	3 to 5		−48.5	90 at 4 and 25 °C	[64]
		20.8		−25.3	NA	[65]

^1^ EE = encapsulation efficiency, NPs = nanoparticles, SLNPs = solid lipid nanoparticles, NA = data not available and NLCs = nanostructured lipid carriers.

**Table 2:** The advantages and disadvantages of nanosystems for encapsulation of carotenoids ^1^.

Nanosystem	Advantages and Disadvantages	References
Nanoemulsions	**Advantages** High optical clarity and enhanced physical stability Small-sized particles with improved bioavailability and absorption Increased solubility of lipophilic compounds Rapid and efficient penetration of the compound Energy efficient method **Disadvantages** Use of large surfactant and co-surfactant Low storage and chemical stability Limited solubility for high melting substances Bio-toxicity of the carrier	[2,5]
Polymeric/biopolymeric NPs	**Advantages** High stability and EE Easy biodegradability and high bioavailability Controlled release, drug targeting and the enhanced cellular uptake Low cost **Disadvantages** Irritation after administration Low storage stability	[3,5]
Nanoliposomes/liposomes	**Advantages** Less toxicity Increased stability, efficiency and pharmacokinetic effects **Disadvantages** Low solubility, short half-life and low EE Difficult to control size of liposomes Less reproducibility High cost ingredients Poor resistance to gastrointestinal enzymes and at low pH	[5,9]
SLNPs	**Advantages** High possibility to encapsulate lipophilic and hydrophilic compounds No use of organic solvents Easy scale-up process High membrane permeability of liposomes and the ability of biopolymer NPs for controlled release High bioactive absorption and easy biodegradability Lack of biotoxicity **Disadvantages** Low EE and stability Presence of others colloidal structures Polymorphic transitions may result in expulsion of bioactive compounds Conformational modification of the lipid NPs	[2,5,9]
NLCs	**Advantages** High EE and stability Controlled release Simple preparation methods with controlled particle size High possibility for scale-up **Disadvantages** Cytotoxic effect Irritation and sensitizing action of surfactants	[2,5]
Supercritical fluid-based NPs	**Advantages** Scalable, green, nontoxic and economical Good particle size with controlled particle morphology High production yield and EE Homogeneous drug distribution Reduced isomerization and thermal degradation of heat labile compounds Solvent can be easily eliminated from food matrix Minimizes harmful chemical residues Low-temperature operation Produces solvent-free and homogenous products Single-step processing method **Disadvantages** Poor solubility of solutes in SCF CO_2_ Size of particles cannot be controlled	[2,9,66]
Metal/metal oxide-based NPs and hybrid nanocomposites	**Advantages** No toxic solvent required Great plasma absorption Target site delivery High surface area Cost-effective High uniformity in shape, size and branch length **Disadvantages** Particles instability Toxic, carcinogenic and cause irritation Less reproducibility of the processes Low possibility for scale-up	[67,68]

^1^ EE = encapsulation efficiency, NPs = nanoparticles, SLNPs = solid lipid nanoparticles and NLCs = nanostructured lipid carriers.

## Abbreviations

ABTS	2,2′-Azino-bis (3-ethylbenzthiazoline-6-sulfonic acid)
BC-CR NEs	β-carotene enriched nanoemulsions prepared from *Citrus reticulate*
BC-MC/BO NEs	β-carotene enriched microbial carotenoids (*Microbacterium* sp.) and buriti oil nanoemulsions
CSZ-NPs	Caseinate-stabilized zein nanoparticles
DPPH	2,2-diphenyl-1-picrylhydrazyl
EE	Encapsulation efficiency
ES-LU-NFs	Electrospun lutein-loaded nanofibers
FX-CH-GL NGs	Fucoxanthin-loaded chitosan-glycolipid hybrid nanogels
IC_50_	Half maximal inhibitory concentration
LGOSNPs	Lycopene-reduced graphene oxide-silver nanoparticles
LN-GNPs	Lycopene nanoemulsions carrying gold nanoparticles
NCs	Nanocapsules
NEs	Nanoemulsions
NFs	Nanofibers
NLs	Nanoliposomes
NLCs	Nanostructured lipid carriers
NPs	Nanoparticles
OEGCG-lycopene NPs	Oligomerized (-) epigallocatechin-3-O-gallate-lycopene nanoparticles
o/w	Oil-in-water
PCL	Poly-ε-caprolactone
PEG	Polyethylene glycol
PLGA	Poly(lactide-co-glycolide)
PLL	Poly-L-lysine
PVA	Polyvinyl alcohol
PVP/Z-AX NPs	Z-astaxanthin nanoparticles with polyvinylpyrrolidone
SEDS	Solution enhanced dispersion by supercritical fluids
SLNPs	Solid-lipid nanoparticles
w/o	Water-in-oil
ZP	Zeta potential
ZX-CE-LB NEs	Zeaxanthin-rich carotenoid extract from *Lycium barbarum L.*
